# A Defined Medium for Cultivation and Exometabolite Profiling of Soil Bacteria

**DOI:** 10.3389/fmicb.2022.855331

**Published:** 2022-05-25

**Authors:** Markus de Raad, Yifan V. Li, Jennifer V. Kuehl, Peter F. Andeer, Suzanne M. Kosina, Andrew Hendrickson, Nicholas R. Saichek, Amber N. Golini, La Zhen Han, Ying Wang, Benjamin P. Bowen, Adam M. Deutschbauer, Adam P. Arkin, Romy Chakraborty, Trent R. Northen

**Affiliations:** ^1^Lawrence Berkeley National Laboratory, Environmental Genomics and Systems Biology Division, Berkeley, CA, United States; ^2^Earth and Environmental Sciences Area, Lawrence Berkeley National Laboratory, Berkeley, CA, United States; ^3^Department of Bioengineering, University of California, Berkeley, Berkeley, CA, United States; ^4^Lawrence Berkeley National Laboratory, Joint Genome Institute, Berkeley, CA, United States

**Keywords:** exometabolomics, liquid chromatography mass spectrometry, defined media, soil bacteria, R2A

## Abstract

Exometabolomics is an approach to assess how microorganisms alter, or react to their environments through the depletion and production of metabolites. It allows the examination of how soil microbes transform the small molecule metabolites within their environment, which can be used to study resource competition and cross-feeding. This approach is most powerful when used with defined media that enable tracking of all metabolites. However, microbial growth media have traditionally been developed for the isolation and growth of microorganisms but not metabolite utilization profiling through Liquid Chromatography Tandem Mass Spectrometry (LC-MS/MS). Here, we describe the construction of a defined medium, the Northen Lab Defined Medium (NLDM), that not only supports the growth of diverse soil bacteria but also is defined and therefore suited for exometabolomic experiments. Metabolites included in NLDM were selected based on their presence in R2A medium and soil, elemental stoichiometry requirements, as well as knowledge of metabolite usage by different bacteria. We found that NLDM supported the growth of 108 of the 110 phylogenetically diverse (spanning 36 different families) soil bacterial isolates tested and all of its metabolites were trackable through LC–MS/MS analysis. These results demonstrate the viability and utility of the constructed NLDM medium for growing and characterizing diverse microbial isolates and communities.

## Introduction

Soil microbes are important as they carry out key ecosystem processes, including the cycling of carbon and other nutrients. Exometabolomics is an approach to determine the metabolites produced or depleted in a given environment (Allen et al., 2003). It enables the direct examination of how soil microbes transform and or synthesize the small molecule metabolites within their environment, providing new insights into resource competition and cross-feeding (Swenson et al., 2018). Detecting the uptake and release of metabolites by a given microorganism or microbial community is done by comparing inoculated vs. uninoculated growth media. For this, a culture medium is needed that supports both the culturing and exometabolomics of bacteria.

A frequently used complex growth medium for isolation and culturing soil bacteria is Reasoner’s 2A (R2A) (Reasoner and Geldreich, 1985). Although R2A was not designed to resemble the soil environment, it has been found to support the growth of a wide variety of soil microbes (Marteinsson et al., 2015; Nunes da Rocha et al., 2015; Nishioka et al., 2016; Nguyen et al., 2018; Chaudhary et al., 2019; Xian et al., 2020). For example, a phylogenetically diverse range of bacteria from the deep subsurface, permafrost, and desert soil crust were isolated and cultured using R2A (Zlatkin et al., 1996; Zhang et al., 2013; Nunes da Rocha et al., 2015). Another form of complex media are soil extracts, which contain solubilized organic and inorganic matter from soil (Liebeke et al., 2009; Nguyen et al., 2018) which are the most ecologically relevant. Similar to soil extracts, metabolites released from other soil bacteria into the environment may be growth-stimulatory and media can be supplemented with spent culture supernatants or cell-free extracts derived from soil isolates (Kim et al., 2008; Baran et al., 2011).

While virtually any growth media can be used for exometabolite profiling, defined media are desirable in that all the metabolites can be accounted for and the media can be made reproducibly. Complex media contain components that are derived from complex organisms (e.g., yeast extract) that can vary in relative composition between batches. Using a defined medium also facilitates detection of microbial secreted products. Defined media designed for isolation often have a single substrate or nutrient source to select for specific organisms, which can be a complex substrate (Davis et al., 2005; Trinh et al., 2019). Some defined media contain a synthetic carbon mixture based on soil extract or cell extracts (Liebeke et al., 2009; Baran et al., 2015). Previously, we reported the development of a defined medium based on water soluble soil metabolites from saprolite soil (Jenkins et al., 2017). Although it was successfully used for exometabolomic profiling, only half as many isolates tested grew in it vs. R2A.

The goal of this study is to design a defined medium, the Northen Lab Defined Medium (NLDM), that supports the growth of a wide range of diverse soil bacteria and allows in depth exometabolomic profiling. Metabolites to be included were selected on the basis of their presence in R2A, their presence in soil, and their usage across a diverse set of bacteria based on existing exometabolomic data (Kosina et al., 2018). The relative abundance of the selected metabolites was based on the knowledge of elemental stoichiometries for bacterial growth and other culture media (Cleveland and Liptzin, 2007). We examined the growth of a panel of 110 phylogenetically diverse isolates from the Oak Ridge Field Research Center (ORFRC) for growth in NLDM vs. R2A and found that the two media supported the growth of comparable numbers of isolates. A subset of these isolates were characterized using exometabolite profiling on NLDM which revealed a high degree of phylogenetic niche conservatism for substrate use.

## Materials and Methods

### Defined Media Composition

All individual chemicals were purchased from Sigma Aldrich (St. Louis, MO, United States) except for sn-glycero-3-Phosphocholine (Cayman Chemical, Ann Arbor, MI, United States). Wolfe’s Vitamin supplement (MD-VS™) and Wolfe’s Trace Mineral supplement (MD-TMS™) were purchased from ATCC (Manassas, VA, United States). NLDM was prepared by adding the 64 metabolites to a base medium composed of 1x Wolfe’s mineral and 1x Wolfe’s vitamin solutions, potassium phosphate, sodium phosphate, calcium chloride, magnesium sulfate, and ammonium chloride (Supplementary Table 1). The pH of NLDM was adjusted to 7.0–7.2 and was sterilized using a 0.22 μm PES filter. A recipe for preparation of NLDM can be found in Supplementary Table 2.

### Growth of 110 Phylogenetically Diverse Oak Ridge Field Research Center Isolates

**FIGURE 1:**
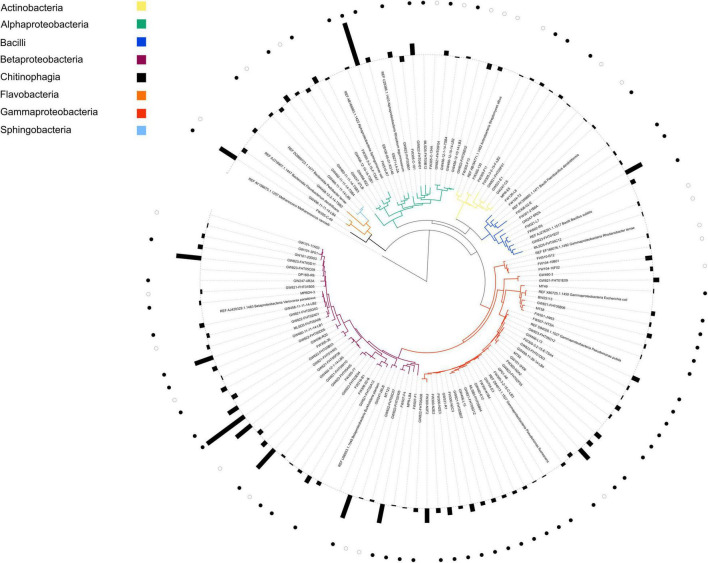
Phylogenetic tree of all isolates with corresponding ratio of growth in NLDM and R2A. Label colors indicate the phylogenetic origin of each isolate by class. REF indicates reference organism and were inserted for guidance and not used in this study. Bars on the outer circle indicate the average (*n* = 2 or *n* = 3) log2 ratio of the growth (maximum OD_600_) of each isolate grown in NLDM and R2A. Ratios > 0 indicate that growth on NLDM was higher than in R2A and ratios < 0 indicate that growth in R2A was higher than in NLDM. Closed circles indicate that growth was significantly (*P* < 0.05) higher in NLDM compared to R2A; open circles indicate that growth was significantly (*P* < 0.05) higher in R2A compared to NLDM (pairwise *t*-test). All growth data (OD_600_ values over time for all isolates) can be found in Supplementary Tables 7A–C. A phylogenetic tree including all reference and guide sequences is shown in Supplementary Figure 1.

NLDM was compared to R2A medium at 1x concentration (Tecknova, Hollister CA) for their ability to support the growth of a broad range of ORFRC isolates, each in duplicate or triplicate. For growth analysis, the 110 phylogenetically diverse isolates (Supplementary Table 3) were revived in 5 mL liquid R2A medium from frozen glycerol stocks or from single colonies from frozen glycerol stocks streaked onto R2A plates. Aliquots from overnight cultures were washed twice with PBS (Sigma Aldrich) by centrifugation at 7,000 × g and diluted with the test medium, either R2A or NLDM, to an OD_600_ of 0.1 prior to inoculation into 96-well plates (Corning CLS3370, Sigma Aldrich). For each fresh medium, 36 μL of washed starter culture (or 36 μL of sterile medium for uninoculated media controls) was added to 144 μL test medium, either R2A or NLDM, (for a final OD_600_ of 0.02) and plates were incubated under aerobic conditions for 24–48 h at 27°C, and shaken at 250 rpm. Growth data were collected by measuring OD_600_ on microplate readers at 15 min intervals. Growth was defined as an increase of 0.05 or greater from the first time point (max OD_600_–initial OD_600_) after subtraction of the uninoculated media control. The significance between growth in NLDM vs. R2A for all isolates was analyzed with Python pingouin 0.3.11 pairwise *T*-tests corrected *p*-values including the interaction between isolate and medium and taxonomic class and medium in a separate analysis using Benjamini/Hochberg false discovery rate correction. A phylogenetic tree was constructed from isolate 16S rRNA gene sequences. The16S rRNA gene of the isolates were PCR amplified using primers 27F and 1492R and Sanger sequencing was performed directly on the PCR products. The 16S rRNA gene sequences were manually end-trimmed to remove ambiguities. The shortest trimmed sequence from each phylogentic family was used to recruit guide sequences from the SILVA LTP and RDP databases. A total of 98 guide sequences were added to the global alignment along with 13 “References” sequences composed of 16S rRNA gene sequences of well-studied and characterized isolates to help orient the reader (marked as “REF” in Figure 1; see Supplementary Information for complete list of FASTA sequences). The sequence alignment was performed in MAFFT (version 7) using the L-INS-i iterative refinement method (Katoh and Standley, 2013). The tree was prepared using the FastTree 2 software package with the Generalized Time-Reversible (GTR) model and optimized Gamma20 likelihood with the “pseudocounts” flag for uneven sequence length (Price et al., 2010; Supplementary Figure 1). For clarity, the resulting tree was pruned of guide sequences within TreeGraph 2 prior to visualization which was imported and displayed using the ITOL software (Letunic and Bork, 2021).

### Exometabolomics Sample Preparation

Triplicate 1.2 mL cultures (including medium controls) of 30 different isolates were cultured in NLDM at 30°C using 24 well plates (same inoculation technique described in the previous section). A single time point was collected from individual wells, each at 24 h. A culture fraction of 1 mL was centrifuged at 7,000 × g for 5 min at 4°C and 0.5 mL of the supernatant was collected. The supernatants were then frozen at –80°C, lyophilized to dryness and resuspended in 250 μL methanol containing internal standards (Supplementary Table 4). The resuspended samples were filtered through 0.2 μm modified nylon membrane centrifugal filters (Pall Corporation, Port Washington, NY, United States) for 2 min at 5,000 × g and analyzed as described in the Liquid Chromatography Tandem Mass Spectrometry (LC-MS/MS). section.

### Liquid Chromatography Tandem Mass Spectrometry (LC-MS/MS) Analysis and Metabolite Identification and Statistical Analysis

Metabolites in the NLDM were chromatographically separated using hydrophilic interaction liquid chromatography (HILIC) and detected by high resolution tandem mass spectrometry. Analyses were performed using an InfinityLab Poroshell 120 HILIC-Z column (Agilent, Santa Clare, CA, United States) on an Agilent 1,290 stack connected to a Q-Exactive Hybrid Quadrupole-Orbitrap Mass Spectrometer (Thermo Fisher Scientific, Waltham, MA, United States) equipped with a Heated Electrospray Ionization (HESI-II) source probe. Separation, ionization, fragmentation and data acquisition parameters are specified in Supplementary Table 4. Briefly, metabolites were separated by gradient elution followed by MS1 and data dependent (top 2 most abundant MS1 ions not previously fragmented in last 7 s) MS2 collection; targeted data analysis was performed by comparison of sample peaks to a library of analytical standards analyzed under the same conditions. Three parameters were compared: matching m/z, retention time and fragmentation spectra using Metabolite Atlas^1^ (Bowen and Northen, 2010; Yao et al., 2015). Metabolite background signals detected in the extraction blanks were subtracted from the experimental sample peak heights/areas. Metabolite peak heights and peak areas were normalized by setting the maximum peak height or peak area detected in NLDM uninoculated control samples to 100%. Normalized metabolite feature peak heights or peak areas were used for relative abundance comparisons. Hierarchical clustering analysis with a Bray–Curtis dissimilarity matrix was performed with the Python 3.7.4 Seaborn 0.9.0 package. The significance between control medium and isolate metabolic profiles was analyzed with the Python Scipy version 1.6.3 Ranksums test.

Spearman correlation coefficients between metabolite fold-change patterns were calculated with Pengouin version 0.3.11 pairwise correlations and Bonferroni correction to the *p*-values. Exometabolomic profile distances used are the Euclidean pairwise distances of the isolate metabolic profiles relative to uninoculated media (log 2 normalized). Corresponding phylogenetic distances were generated by first aligning trimmed 16S rRNA gene sequences from the isolates in the MAFFT software package (version 7) using the L-INS-i iterative refinement method and followed by the generation of a distance matrix using the FastTree 2 software package using the Generalized Time-Reversible (GTR) model and optimized Gamma20 likelihood (Price et al., 2010; Katoh and Standley, 2013). The distance matrix generated for the phylogenetic tree (e.g., distance between isolates on the phylogentic tree) were used on the x-axis. Raw data and targeted outputs have been deposited in the JGI Genome Portal under FD ID 1354404 (Nordberg et al., 2014).

Feature-Based Molecular Networking (FBMN) was performed using MZmine 2 and GNPS. A molecular network was created with the Feature-Based Molecular Networking (FBMN) workflow on GNPS^2^ (Wang et al., 2016; Nothias et al., 2020). The mass spectrometry data were first processed with MZMINE2 (see Supplementary Xml File for detailed parameters) and the results were exported to GNPS for FBMN analysis. The data was filtered by removing all MS/MS fragment ions within ± 17 Da of the precursor m/z. MS/MS spectra were window filtered by choosing only the top 6 fragment ions in the ± 50 Da window throughout the spectrum. The precursor ion mass tolerance was set to 0.01 Da and the MS/MS fragment ion tolerance to 0.02 Da. A molecular network was then created where edges were filtered to have a cosine score above 0.70 and more than 3 matched peaks. Further, edges between two nodes were kept in the network if and only if each of the nodes appeared in each others respective top 10 most similar nodes. Finally, the maximum size of a molecular family was set to 0, and the lowest scoring edges were removed from molecular families until the molecular family size was below this threshold. The spectra in the network were then searched against GNPS spectral libraries (Horai et al., 2010; Wang et al., 2016). The library spectra were filtered in the same manner as the input data. All matches kept between network spectra and library spectra were required to have a score above 0.4 and at least 3 matched peaks. The molecular networks were visualized using Cytoscape software (Shannon et al., 2003). Features observed in 3 or less (3 out 18 = 16.7%) NLDM uninoculated control samples were colored green to yellow in the networks based on the ratio (0.01–1) of the number of isolate spent medium samples the feature was detected in divided by the total number of isolate spent medium samples. Only features with 10× higher peak heights than in extraction blanks were selected for analysis.

## Results

To balance microbial growth, metabolite diversity, and compositional simplicity (avoiding an excessive number of metabolites), NLDM is primarily composed of the metabolite composition of R2A supplemented with Wolfe’s minerals and Wolfe’s vitamins (Supplementary Table 1). Additional included metabolites were either detected in soil obtained from the same ORFRC site where the microbes used in this study were isolated from or in soil organic matter analyzed in other studies (Supplementary Table 5; Swenson et al., 2015; Jenkins et al., 2017; Sasse et al., 2019). In addition, the exometabolomic assertion repository, Web of Microbes, was used to assess if the included metabolites are commonly consumed by bacteria (Kosina et al., 2018). Metabolite concentrations in NLDM were adjusted to (1) have the C:N ratio consistent with the soil microbial biomass and (2) mimic compound class ratios found in R2A (Cleveland and Liptzin, 2007).

### Metabolite Selection

Except for glucose and pyruvic acid, R2A is a complex and undefined metabolite mixture, primarily based on yeast extract, casamino acids, soluble starch, and proteose peptone (Reasoner and Geldreich, 1985). To identify the small molecules from these complex components, we previously analyzed R2A medium using LC-MS/MS (Supplementary Table 5; Jenkins et al., 2017; Kosina et al., 2018). Through these efforts, a number of metabolites present in R2A were identified. These included most of the standard amino acids, all 5 standard nucleobases, and 3 standard ribonucleosides. Based on these findings, we included all 20 standard amino acids, the 5 standard nucleobases, and the 5 standard ribonucleosides in NLDM. In addition, the amino acid citrulline, the nucleobases and ribonucleosides xanthine, hypoxanthine, inosine and xanthosine were included in NLDM based on their presence in R2A.

Primary energy sources in R2A are the sugar glucose and the glucose polymer starch; the latter is too large for small molecule LC-MS/MS detection. As a result, we included glucose plus two additional sugars detected in R2A, the dihexose trehalose and the sugar alcohol myo-inositol. In addition, to increase substrate diversity and assess additional metabolic pathways, we also included the pentose xylose, the dihexose sucrose, and the amino sugars N-acetyl-glucosamine and n-acetylmuramic acid, which are commonly found in soils (Glaser et al., 2004; Gunina and Kuzyakov, 2015; Sasse et al., 2019; Ni et al., 2020). Pyruvic acid is another major defined energy source in R2A medium. To capture organic acids as potential energy sources for bacteria pyruvic acid was included along with 5 other common organic acids detected in R2A medium and/or in soil, namely lactic acid, malic acid, citric acid, succinic acid and α-ketoglutaric acid.

Other metabolites were selected for NLDM based on the analysis of R2A medium and/or soil. In total 64 metabolites were selected to be included in NLDM. We decided to include spermidine even though it was not detected in R2A or soil because polyamines are “essential” cofactors and because another polyamine, A-acetylputrescine, has been detected in R2A medium (Xavier et al., 2017).

### Mining Existing Exometabolite Data for Metabolite Usage

After the formulation of NLDM, we checked if the selected metabolites in the environment/medium would be reduced after inoculation with the microbes. To do this, we analyzed the decreased abundance of the selected metabolites by microbes using existing exometabolomic data collected in Web of Microbes (Kosina et al., 2018). A metabolite was deemed decreased if the metabolite was significantly lower in the presence of a microbe compared to the uninoculated control. Out of the 64 metabolites present in NLDM, 54 were in the Web of Microbes database. All but 4 metabolites, α-ketoglutaric acid, cysteine, cytidine and uridine, were decreased by at least 1 microbe (Supplementary Table 6).

### Northen Lab Defined Medium Formulation

The quantitative formulation of the 64 selected metabolites was based on the amount of organic carbon (C) and nitrogen (N) in R2A (Kim et al., 2019). We divided all 64 metabolites in NLDM into 4 different groups: sugars, organic acids, amino acids, and other metabolites (Supplementary Table 1). Metabolites within each group were assigned fixed equimolar concentrations and the total organic C and N was calculated (Table 1). This yielded a C:N ratio of 9:1, which is similar to R2A (8:1) and the soil microbial biomass ratio (9:1) [based on the atomic C:N:P ratios in the soil microbial biomass (60:7:1)] (Cleveland and Liptzin, 2007). As for the salts, NLDM contains 5 mM phosphate, 1 mM ammonium, 2 mM sodium, 7 mM potassium, 1 mM magnesium, 1 mM sulfur, 1 mM calcium, and 2 mM chloride (Supplementary Table 1). NLDM is supplemented with 1x Wolfe’s vitamins and 1x Wolfe’s minerals (Atlas, 2004).

**TABLE 1:** Breakdown of organic C and N in NLDM.

	μM of each metabolite	# of compounds	C (mg/L)	*N* (mg/L)
Sugars	875	7	578	12
Organic acids	525	7	195	22
Amino acids	175	20	225	71
All other metabolites	17.5	30	43	18
	Total	64	1,042	123

### Comparable Growth Is Observed on Northen Lab Defined Medium and R2A Regardless of Phylogeny

We selected 110 bacterial isolates from the same ORFRC field site for this study and compared the growth of the isolates in both NLDM and R2A. Notably, most isolations (35%) were made using R2A (Supplementary Table 3; Thorgersen et al., 2015; Hemme et al., 2016; Liu et al., 2018; Price et al., 2018; Carlson et al., 2019; Vuono et al., 2019; Lui et al., 2021). It was found that only 2 out of the 110 isolates did not display significant growth in NLDM, whereas 6 out of the 110 isolates did not display significant growth in R2A medium (Figure 1 and Supplementary Tables 3, 7A–C). The overall growth (as measured as the highest observed OD_600_ minus starting OD_600_) was similar for R2A and NLDM, except for beta- and gamma proteobacteria which displayed significantly better growth in NLDM than on R2A (Supplementary Figure 2). The lag phases and growth rates were similar for R2A and NLDM, except for betaproteobacteria which displayed significantly shorter lag phase in NLDM than on R2A (Supplementary Figures 3, 4 and Supplementary Table 8).

### Exometabolite Profiling Reveals Phylogenetic Niche Conservatism in Substrate Use

**FIGURE 2:**
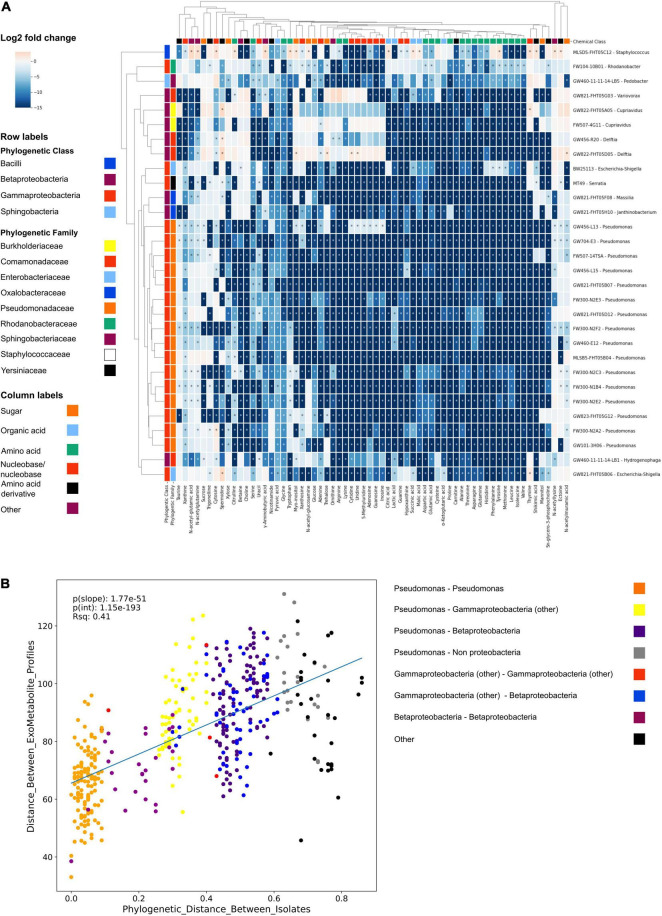
NLDM metabolite utilization patterns by soil isolates. **(A)** NLDM metabolites utilization after 24 h displayed as the log2 of the average normalized peak height or peak area relative to the medium control in a clustering heatmap. *n* = 3 for each isolate. Rows are colored according to phylogenetic class and family level. *Indicates *P* < 0.05 (Ranksum). **(B)** Regression analysis comparing the phylogenetic distance vs. distance between the hierarchical clustering of the exometabolite profiles.

We next used the NLDM to investigate the substrate preferences of 30 isolates using LC-MS/MS. Using hydrophilic interaction liquid chromatography (HILIC) LC-MS/MS, all metabolites were detected in the full medium formulation (Supplementary Table 9). All 64 metabolites from the NLDM medium were utilized by at least 1 isolate after 24 h (compared to medium control using ranksum, *P* < 0.05). *Pseudomonas* FW300-N2F2 decreased the abundance of the most metabolites (63 significantly decreased in abundance relative to media control) whereas *Cupravidius* GW822-FHT05A05 resulted in the fewest metabolites decreased in abundance (34) (Figure 2A). Some NLDM metabolites were excreted by the isolates, as their abundance significantly increased. Feature-based molecular networking indicated that the isolates excreted metabolites not present in NLDM (Supplementary Figure 5). Interestingly, we observed clear differences in metabolite depletion between isolates and between metabolite classes (Figure 2A). Specifically, hierarchical clustering revealed that metabolite utilization is conserved with phylogeny. Correlation analysis of metabolite utilization revealed that isolates belonging to the same family and genus have significantly similar metabolite usage profiles (*P* < 0.05) (Figure 2B).

## Discussion

The goal of this study was to develop a defined medium suitable for both the cultivation and exometabolite profiling of diverse soil bacteria. The final concentrations of sugars, organic acids, amino acids, and other metabolites included in NLDM as well as the inorganic N from the salts make up a C:N ratio that is consistent with R2A and the “Redfield ratio” for soil bacterial biomass (Cleveland and Liptzin, 2007). Compared to soil bacterial biomass, the phosphate concentration in NLDM is high to ensure that phosphate is not limited during culturing. We acknowledge that the chosen metabolite concentrations do not reflect ecological soil conditions, as metabolite concentrations in soil vary by type, location, among other factors. Like R2A, NLDM is a nutrient rich medium. This can be an issue since it is thought that nutrient rich culture media favor the growth of faster-growing bacteria at the expense of slow-growing species which can also be inhibited by substrate-rich conventional media (Vartoukian et al., 2010; Nunes da Rocha et al., 2015; Overmann et al., 2017; Bartelme et al., 2020).

Compared to our previously reported soil defined medium (SDM), NLDM is richer in terms of total carbon (1,146 mg/L vs. 355 mg/L organic C, for NLDM and SDM, respectively), has a more balanced C:N ratio (9:1 vs. 1:1) and contains a wider selection of metabolites (64 vs. 46 metabolites) (Jenkins et al., 2017). Furthermore, NLDM supported the growth of 9 isolates that did not grow on SDM (Jenkins et al., 2017). We anticipate that NLDM can also be diluted to examine more oligotrophic bacteria; however, the culture volume will need to be increased to provide sufficient signal for LC-MS/MS analyses in exometabolomic experiments.

Although R2A was used to guide the NLDM formulation, most of the metabolites included are also present in several other soil-extract based media (Liebeke et al., 2009; Jenkins et al., 2017; Nguyen et al., 2018; Sasse et al., 2019). The metabolite diversity in NLDM is lower than complex media, which include undefined and variable components such as yeast extract. However, we determined this is a worthwhile tradeoff given that this medium allows for a comprehensive view of metabolite usage using LC-MS/MS. Furthermore, the strength of a defined medium is that it allows to track excreted metabolites and we observed several metabolites in isolate spent medium that were not present in NLDM (Supplementary Figure 2). A major difference between complex media and NLDM is that NLDM contains a single phospholipid whereas other media contain a range of different fatty acids (Kankaanpää et al., 2004; Liebeke et al., 2009). This lack could affect growth as fatty acid synthesis is an energy and material intensive process, and the incorporation of usable exogenous fatty acids saves energy and metabolic building materials (Yao and Rock, 2017). We anticipate that NLDM can be used as a base medium for supplementation with other compounds of interest to increase its relevance to soil and to extend to additional classes of metabolites, for example to include additional lipids.

Analysis of the growth of a diverse group of isolates in NLDM vs. R2A showed comparable growth. We found that 108 out of the 110 bacteria tested grew on NLDM. In fact, 63 of the 110 isolates tested reached a significantly higher maximum OD_600_ on NLDM compared to R2A and all but three of the 25 pseudomonads tested displayed higher growth in NLDM than in R2A. This is interesting because R2A was originally developed to isolate pseudomonads from treated potable water (Reasoner and Geldreich, 1985). The growth rates of the isolates on R2A and NLDM were similar, indicating that the isolates have a similar fitness on both media (Ram et al., 2019). A shorter lag phase was observed for betaproteobacteria grown in NLDM. This could be attributed to the higher concentration of immediately accessible metabolites in NLDM compared to R2A as R2A contains biopolymers which require depolymerization prior to use, resulting in delayed isolate growth (Korem Kohanim et al., 2018).

The application of NLDM to investigate the substrate preferences of 30 isolates revealed clustering by phylogeny, an example of phylogenetic niche conservatism. This has been observed before in similar studies (Bauer et al., 2015; Hester et al., 2019; Fahimipour and Gross, 2020). Notably, all metabolites in NLDM were utilized by at least 1 isolate after 24 h. Interestingly, sugars were among the least depleted chemical classes, with both *Cupriavidus* sp. FW507-4G11 and GW822-FHT05A05 (final OD_600_ of 0.25 and 0.22 after 24 h) not utilizing any of the sugars. In contrast, nucleosides and nucleobases were one of the most utilized classes and used by all isolates. Presumably, this is *via* purine and pyrimidine salvage pathways which are known to be major pathways for bacteria to obtain carbon, energy, and nitrogen for growth (Vogels and Van der Drift, 1976; Nygaard, 1993). Metabolite depletion patterns were similar for closely related isolates, although differences between the individual isolates can be observed (Figure 2A). These observations can be used in future analyses to examine how substrate use relates to the environment of the ORFRC field site from which all the screened isolates originate.

Overall, we find that this NLDM formulation is a useful medium for both bacterial growth and exometabolite profiling. We see many opportunities to alter the formulation to address the needs of specific studies. Certainly, additional metabolites can be included to expand exometabolite profiling and growth assays. The lack of biopolymers in this media compared to soil and R2A may also be a limitation for some organisms. We elected not to include biopolymers, which can confound exometabolite analysis, since the resulting monomers/oligomers are both consumed by microbes and produced by exoenzymes. NLDM could be used in combination with biopolymers for growth and isolation when exometabolite profiling is not required. The 1x concentration of NLDM, while designed to mimic 1x R2A and represent soil composition, is dramatically richer than soil dissolved organic carbon and nitrogen, which typically range between 0.42–372.1 mg C/L and 0.025–10 mg N/L, respectively (Ros et al., 2009; Langeveld et al., 2020). NLDM was not designed to approximate the carbon, nitrogen or metabolite concentrations in soil, which vary considerably. Dilution of 1 or more of the metabolite groups will bring the carbon and nitrogen concentrations closer to soil, but this can hinder both exometabolomic profiling and bacterial growth. Furthermore, evaluating NLDM for microbial isolation is a promising future direction that is outside of the scope of the current work.

## Conclusion

This study describes a new defined medium, NLDM, that is based on the metabolite composition of R2A and soil, metabolite usage by microbes, and biologically relevant elemental stoichiometry of these compounds. NLDM was found to support the growth of 108 out of 110 phylogenetically diverse isolates tested with comparable biomass yields and growth rates. An exometabolomics study using NLDM and 30 different isolates showed that all metabolites in NLDM were significantly depleted by at least one isolate and revealed phylogenetic niche conservatism in substrate use. We anticipate that the NLDM medium will enable the examination of microbial substrate utilization for a broad range of isolates both directly and through dilution and amendments. We speculate that this media may have additional value in supporting microbial isolations and additional types of microbial characterization.

## Data Availability Statement

The datasets presented in this study can be found in online repositories. The names of the repository/repositories and accession number(s) can be found below: The raw LC/MS data and targeted outputs for this study can be found in the JGI Genome Portal under FD ID: 1354404 (https://genome.jgi.doe.gov/portal/pages/dynamicOrganismDownload.jsf?organism=202Nortabolomics_2_FD). Exometabolite profiling results can be found in Web of Microbes (http://www.webofmicrobes.org/).

## Author Contributions

MR, PA, SK, NS, YW, BB, and TN designed the NLDM medium. MR and TN designed the study and the experiments. MR, YL, JK, AH, AA, and RC performed isolate growth screen. MR and AD performed exometabolomic profiling. AG and LH performed sample preparation for LC/MS analysis. AG, LH, and SK acquired all mass spectrometry data. MR, PA, and BB analyzed all growth and mass spectrometry data. MR and TN wrote the manuscript, with contributions from all co-authors. All authors contributed to the article and approved the submitted version.

## Conflict of Interest

The authors declare that the research was conducted in the absence of any commercial or financial relationships that could be construed as a potential conflict of interest.

## Publisher’s Note

All claims expressed in this article are solely those of the authors and do not necessarily represent those of their affiliated organizations, or those of the publisher, the editors and the reviewers. Any product that may be evaluated in this article, or claim that may be made by its manufacturer, is not guaranteed or endorsed by the publisher.

## Footnotes

1
https://github.com/biorack/metatlas

2
https://gnps.ucsd.edu

## Supplementary Material

Supplementary Figure 1 Phylogenetic tree of isolate sequences, guide sequences and reference sequences. The phylogenetic tree forms the basis for the phylogenetic tree in Figure 1. Label colors indicate the phylogenetic origin of each sequence by class. REF and Guide indicates reference organism and were inserted for guidance and not used in this study. A complete list of the FASTA sequences can be found in Supplementary Information.

Supplementary Figure 2 Growth of isolates grown in R2A and NLDM. **(A)** Average (*n* = 2 or *n* = 3) growth (as measured as the highest observed OD_600_ minus starting OD_600_) of each isolate grown on NLDM (in blue) and R2A (yellow). Black bars represent the average for each medium type. **(B)** Average (*n* = 2 or *n* = 3) growth (as measured as the highest observed OD_600_ minus starting OD_600_) of each isolate grown on NLDM (in blue) and R2A (yellow) per phylogenetic class. *Indicate that growth was significantly (*P* < 0.05) higher in NLDM compared to R2A (pairwise *t*-test).

Supplementary Figure 3 Lag phase of isolates grown in R2A and NLDM. **(A)** Average (*n* = 2 or *n* = 3) lag phase of each isolate grown on NLDM (in blue) and R2A (yellow). Black bars represent the average for each medium type. **(B)** Average (*n* = 2 or *n* = 3) lag phase of each isolate grown on NLDM (in blue) and R2A (yellow) per phylogenetic class. *Indicate that the lag phase was significantly (*P* < 0.05) shorter in NLDM compared to R2A (pairwise *t*-test).

Supplementary Figure 4 Growth rates of isolates grown in R2A and NLDM. **(A)** Average (*n* = 2 or *n* = 3) growth rate of each isolate grown on NLDM (in blue) and R2A (yellow). Black bars represent the average for each medium type. **(B)** Average (*n* = 2 or *n* = 3) growth rates of each isolate grown on NLDM (in blue) and R2A (yellow) per phylogenetic class.

Supplementary Figure 5 Feature-based molecular networking. Identification of “produced” metabolites by comparing features observed in uninoculated medium vs. in isolate spent medium. Feature-based molecular networks with features detected in positive **(A)** and negative **(B)** polarity were colored to illustrate signals coming from metabolites produced by the isolates in this study. Features observed in less than 16% of NLDM uninoculated controls were colored from dark to light based on the ratio (0.01–1) of the number of samples of isolate spent medium the feature was observed in divided by the total number of isolate spent medium samples, with a ratio of 1 indicating that the feature was observed in all isolate spent medium samples.
