# Supplementary material for: A Defined Medium for Cultivation and Exometabolite Profiling of Soil Bacteria
Source: Front Microbiol. 2022 May 25;13:855331. doi: 10.3389/fmicb.2022.855331 (PMC9174792; doi:10.3389/fmicb.2022.855331)
Supplement: Supplementary file 2 [file Data_Sheet_2.PDF]

```
<?xml version="1.0" encoding="utf-8"?>
<batch><batchstep
method="net.sf.mzmine.modules.rawdatamethods.rawdataimport.RawDataImpo
rtModule"><parameter name="Raw data file names"><file>/global/project/
projectdirs/metatlas/raw_data/agolini/20201218_LZH-
AG_MdR_ENIGMA_Atlas_20201111rnd1_QE119_Ag683775-924_USHXG01474/2020121
8_LZH-
AG_MdR_ENIGMA_Atlas_20201111rnd1_QE119_Ag683775-924_USHXG01474_POS_MSM
S-v2_0_QC-PrimMet-SOPv3_IR003__015.mzML</file><file>/global/project/
projectdirs/metatlas/raw_data/agolini/20201218_LZH-
AG_MdR_ENIGMA_Atlas_20201111rnd1_QE119_Ag683775-924_USHXG01474/2020121
8_LZH-
AG_MdR_ENIGMA_Atlas_20201111rnd1_QE119_Ag683775-924_USHXG01474_POS_MSM
S-v2_100_NLDM-FHT5-G03-pl5_1_failedinj-2mm-noWBS_022.mzML</
file><file>/global/project/projectdirs/metatlas/raw_data/agolini/
20201218_LZH-
AG_MdR_ENIGMA_Atlas_20201111rnd1_QE119_Ag683775-924_USHXG01474/2020121
8_LZH-
AG_MdR_ENIGMA_Atlas_20201111rnd1_QE119_Ag683775-924_USHXG01474_POS_MSM
S-v2_100_NLDM-FHT5-G03-pl5_1_reinj-postfailed_400.mzML</file><file>/
global/project/projectdirs/metatlas/raw_data/agolini/20201218_LZH-
AG_MdR_ENIGMA_Atlas_20201111rnd1_QE119_Ag683775-924_USHXG01474/2020121
8_LZH-
AG_MdR_ENIGMA_Atlas_20201111rnd1_QE119_Ag683775-924_USHXG01474_POS_MSM
S-v2_101_NLDM-FHT5-G03-pl5_2__267.mzML</file><file>/global/project/
projectdirs/metatlas/raw_data/agolini/20201218_LZH-
AG_MdR_ENIGMA_Atlas_20201111rnd1_QE119_Ag683775-924_USHXG01474/2020121
8_LZH-
AG_MdR_ENIGMA_Atlas_20201111rnd1_QE119_Ag683775-924_USHXG01474_POS_MSM
S-v2_102_NLDM-FHT5-G03-pl5_3__085.mzML</file><file>/global/project/
projectdirs/metatlas/raw_data/agolini/20201218_LZH-
AG_MdR_ENIGMA_Atlas_20201111rnd1_QE119_Ag683775-924_USHXG01474/2020121
8_LZH-
AG_MdR_ENIGMA_Atlas_20201111rnd1_QE119_Ag683775-924_USHXG01474_POS_MSM
S-v2_103_NLDM-FHT5-G12-pl5_1__100.mzML</file><file>/global/project/
projectdirs/metatlas/raw_data/agolini/20201218_LZH-
AG_MdR_ENIGMA_Atlas_20201111rnd1_QE119_Ag683775-924_USHXG01474/2020121
8_LZH-
AG_MdR_ENIGMA_Atlas_20201111rnd1_QE119_Ag683775-924_USHXG01474_POS_MSM
S-v2_104_NLDM-FHT5-G12-pl5_2__298.mzML</file><file>/global/project/
projectdirs/metatlas/raw_data/agolini/20201218_LZH-
AG_MdR_ENIGMA_Atlas_20201111rnd1_QE119_Ag683775-924_USHXG01474/2020121
8_LZH-
AG_MdR_ENIGMA_Atlas_20201111rnd1_QE119_Ag683775-924_USHXG01474_POS_MSM
S-v2_105_NLDM-FHT5-G12-pl5_3__238.mzML</file><file>/global/project/
projectdirs/metatlas/raw_data/agolini/20201218_LZH-
AG_MdR_ENIGMA_Atlas_20201111rnd1_QE119_Ag683775-924_USHXG01474/2020121
8_LZH-
AG_MdR_ENIGMA_Atlas_20201111rnd1_QE119_Ag683775-924_USHXG01474_POS_MSM
S-v2_106_NLDM-FHT5-H10-pl5_1__088.mzML</file><file>/global/project/
```

projectdirs/metatlas/raw_data/agolini/20201218_LZH-
AG_MdR_ENIGMA_Atlas_20201111rnd1_QE119_Ag683775-924_USHXG01474/2020121
8_LZH-
AG_MdR_ENIGMA_Atlas_20201111rnd1_QE119_Ag683775-924_USHXG01474_POS_MSM
S-v2_107_NLDM-FHT5-H10-pl5_2__370.mzML</file><file>/global/project/
projectdirs/metatlas/raw_data/agolini/20201218_LZH-
AG_MdR_ENIGMA_Atlas_20201111rnd1_QE119_Ag683775-924_USHXG01474/2020121
8_LZH-
AG_MdR_ENIGMA_Atlas_20201111rnd1_QE119_Ag683775-924_USHXG01474_POS_MSM
S-v2_108_NLDM-FHT5-H10-pl5_3__307.mzML</file><file>/global/project/
projectdirs/metatlas/raw_data/agolini/20201218_LZH-
AG_MdR_ENIGMA_Atlas_20201111rnd1_QE119_Ag683775-924_USHXG01474/2020121
8_LZH-
AG_MdR_ENIGMA_Atlas_20201111rnd1_QE119_Ag683775-924_USHXG01474_POS_MSM
S-v2_109_NLDM-TxCtrl-pl5_1__061.mzML</file><file>/global/project/
projectdirs/metatlas/raw_data/agolini/20201218_LZH-
AG_MdR_ENIGMA_Atlas_20201111rnd1_QE119_Ag683775-924_USHXG01474/2020121
8_LZH-
AG_MdR_ENIGMA_Atlas_20201111rnd1_QE119_Ag683775-924_USHXG01474_POS_MSM
S-v2_10_NLDM-AMD440-pl1_1__154.mzML</file><file>/global/project/
projectdirs/metatlas/raw_data/agolini/20201218_LZH-
AG_MdR_ENIGMA_Atlas_20201111rnd1_QE119_Ag683775-924_USHXG01474/2020121
8_LZH-
AG_MdR_ENIGMA_Atlas_20201111rnd1_QE119_Ag683775-924_USHXG01474_POS_MSM
S-v2_110_NLDM-TxCtrl-pl5_2__291.mzML</file><file>/global/project/
projectdirs/metatlas/raw_data/agolini/20201218_LZH-
AG_MdR_ENIGMA_Atlas_20201111rnd1_QE119_Ag683775-924_USHXG01474/2020121
8_LZH-
AG_MdR_ENIGMA_Atlas_20201111rnd1_QE119_Ag683775-924_USHXG01474_POS_MSM
S-v2_111_NLDM-TxCtrl-pl5_3__118.mzML</file><file>/global/project/
projectdirs/metatlas/raw_data/agolini/20201218_LZH-
AG_MdR_ENIGMA_Atlas_20201111rnd1_QE119_Ag683775-924_USHXG01474/2020121
8_LZH-
AG_MdR_ENIGMA_Atlas_20201111rnd1_QE119_Ag683775-924_USHXG01474_POS_MSM
S-v2_112_NA-ExCtrl-NA_1_failedinj-2mm-noWBS_025.mzML</file><file>/
global/project/projectdirs/metatlas/raw_data/agolini/20201218_LZH-
AG_MdR_ENIGMA_Atlas_20201111rnd1_QE119_Ag683775-924_USHXG01474/2020121
8_LZH-
AG_MdR_ENIGMA_Atlas_20201111rnd1_QE119_Ag683775-924_USHXG01474_POS_MSM
S-v2_112_NA-ExCtrl-NA_1_reinj-postfailed_403.mzML</file><file>/global/
project/projectdirs/metatlas/raw_data/agolini/20201218_LZH-
AG_MdR_ENIGMA_Atlas_20201111rnd1_QE119_Ag683775-924_USHXG01474/2020121
8_LZH-
AG_MdR_ENIGMA_Atlas_20201111rnd1_QE119_Ag683775-924_USHXG01474_POS_MSM
S-v2_113_NA-ExCtrl-NA_2__109.mzML</file><file>/global/project/
projectdirs/metatlas/raw_data/agolini/20201218_LZH-
AG_MdR_ENIGMA_Atlas_20201111rnd1_QE119_Ag683775-924_USHXG01474/2020121
8_LZH-
AG_MdR_ENIGMA_Atlas_20201111rnd1_QE119_Ag683775-924_USHXG01474_POS_MSM
S-v2_114_NA-ExCtrl-NA_3__388.mzML</file><file>/global/project/

projectdirs/metatlas/raw_data/agolini/20201218_LZH-
AG_MdR_ENIGMA_Atlas_20201111rnd1_QE119_Ag683775-924_USHXG01474/2020121
8_LZH-
AG_MdR_ENIGMA_Atlas_20201111rnd1_QE119_Ag683775-924_USHXG01474_POS_MSM
S-v2_11_NLDM-AMD440-pl1_2__205.mzML</file><file>/global/project/
projectdirs/metatlas/raw_data/agolini/20201218_LZH-
AG_MdR_ENIGMA_Atlas_20201111rnd1_QE119_Ag683775-924_USHXG01474/2020121
8_LZH-
AG_MdR_ENIGMA_Atlas_20201111rnd1_QE119_Ag683775-924_USHXG01474_POS_MSM
S-v2_12_NLDM-AMD440-pl1_3__394.mzML</file><file>/global/project/
projectdirs/metatlas/raw_data/agolini/20201218_LZH-
AG_MdR_ENIGMA_Atlas_20201111rnd1_QE119_Ag683775-924_USHXG01474/2020121
8_LZH-
AG_MdR_ENIGMA_Atlas_20201111rnd1_QE119_Ag683775-924_USHXG01474_POS_MSM
S-v2_13_NLDM-AMD442-pl1_1__220.mzML</file><file>/global/project/
projectdirs/metatlas/raw_data/agolini/20201218_LZH-
AG_MdR_ENIGMA_Atlas_20201111rnd1_QE119_Ag683775-924_USHXG01474/2020121
8_LZH-
AG_MdR_ENIGMA_Atlas_20201111rnd1_QE119_Ag683775-924_USHXG01474_POS_MSM
S-v2_14_NLDM-AMD442-pl1_2__337.mzML</file><file>/global/project/
projectdirs/metatlas/raw_data/agolini/20201218_LZH-
AG_MdR_ENIGMA_Atlas_20201111rnd1_QE119_Ag683775-924_USHXG01474/2020121
8_LZH-
AG_MdR_ENIGMA_Atlas_20201111rnd1_QE119_Ag683775-924_USHXG01474_POS_MSM
S-v2_15_NLDM-AMD442-pl1_3__229.mzML</file><file>/global/project/
projectdirs/metatlas/raw_data/agolini/20201218_LZH-
AG_MdR_ENIGMA_Atlas_20201111rnd1_QE119_Ag683775-924_USHXG01474/2020121
8_LZH-
AG_MdR_ENIGMA_Atlas_20201111rnd1_QE119_Ag683775-924_USHXG01474_POS_MSM
S-v2_16_NLDM-AMD502-pl1_1__172.mzML</file><file>/global/project/
projectdirs/metatlas/raw_data/agolini/20201218_LZH-
AG_MdR_ENIGMA_Atlas_20201111rnd1_QE119_Ag683775-924_USHXG01474/2020121
8_LZH-
AG_MdR_ENIGMA_Atlas_20201111rnd1_QE119_Ag683775-924_USHXG01474_POS_MSM
S-v2_17_NLDM-AMD502-pl1_2__261.mzML</file><file>/global/project/
projectdirs/metatlas/raw_data/agolini/20201218_LZH-
AG_MdR_ENIGMA_Atlas_20201111rnd1_QE119_Ag683775-924_USHXG01474/2020121
8_LZH-
AG_MdR_ENIGMA_Atlas_20201111rnd1_QE119_Ag683775-924_USHXG01474_POS_MSM
S-v2_18_NLDM-AMD502-pl1_3__043.mzML</file><file>/global/project/
projectdirs/metatlas/raw_data/agolini/20201218_LZH-
AG_MdR_ENIGMA_Atlas_20201111rnd1_QE119_Ag683775-924_USHXG01474/2020121
8_LZH-
AG_MdR_ENIGMA_Atlas_20201111rnd1_QE119_Ag683775-924_USHXG01474_POS_MSM
S-v2_19_NLDM-AMD560-pl1_1__208.mzML</file><file>/global/project/
projectdirs/metatlas/raw_data/agolini/20201218_LZH-
AG_MdR_ENIGMA_Atlas_20201111rnd1_QE119_Ag683775-924_USHXG01474/2020121
8_LZH-
AG_MdR_ENIGMA_Atlas_20201111rnd1_QE119_Ag683775-924_USHXG01474_POS_MSM
S-v2_1_NLDM-AMD35-pl1_1__091.mzML</file><file>/global/project/

projectdirs/metatlas/raw_data/agolini/20201218_LZH-
AG_MdR_ENIGMA_Atlas_20201111rnd1_QE119_Ag683775-924_USHXG01474/2020121
8_LZH-
AG_MdR_ENIGMA_Atlas_20201111rnd1_QE119_Ag683775-924_USHXG01474_POS_MSM
S-v2_20_NLDM-AMD560-pl1_2__136.mzML</file><file>/global/project/
projectdirs/metatlas/raw_data/agolini/20201218_LZH-
AG_MdR_ENIGMA_Atlas_20201111rnd1_QE119_Ag683775-924_USHXG01474/2020121
8_LZH-
AG_MdR_ENIGMA_Atlas_20201111rnd1_QE119_Ag683775-924_USHXG01474_POS_MSM
S-v2_21_NLDM-AMD560-pl1_3__288.mzML</file><file>/global/project/
projectdirs/metatlas/raw_data/agolini/20201218_LZH-
AG_MdR_ENIGMA_Atlas_20201111rnd1_QE119_Ag683775-924_USHXG01474/2020121
8_LZH-
AG_MdR_ENIGMA_Atlas_20201111rnd1_QE119_Ag683775-924_USHXG01474_POS_MSM
S-v2_22_NLDM-TxCtrl-pl1_1__037.mzML</file><file>/global/project/
projectdirs/metatlas/raw_data/agolini/20201218_LZH-
AG_MdR_ENIGMA_Atlas_20201111rnd1_QE119_Ag683775-924_USHXG01474/2020121
8_LZH-
AG_MdR_ENIGMA_Atlas_20201111rnd1_QE119_Ag683775-924_USHXG01474_POS_MSM
S-v2_23_NLDM-TxCtrl-pl1_2__106.mzML</file><file>/global/project/
projectdirs/metatlas/raw_data/agolini/20201218_LZH-
AG_MdR_ENIGMA_Atlas_20201111rnd1_QE119_Ag683775-924_USHXG01474/2020121
8_LZH-
AG_MdR_ENIGMA_Atlas_20201111rnd1_QE119_Ag683775-924_USHXG01474_POS_MSM
S-v2_24_NLDM-TxCtrl-pl1_3__097.mzML</file><file>/global/project/
projectdirs/metatlas/raw_data/agolini/20201218_LZH-
AG_MdR_ENIGMA_Atlas_20201111rnd1_QE119_Ag683775-924_USHXG01474/2020121
8_LZH-
AG_MdR_ENIGMA_Atlas_20201111rnd1_QE119_Ag683775-924_USHXG01474_POS_MSM
S-v2_25_NLDM-AMD892-pl2_1__112.mzML</file><file>/global/project/
projectdirs/metatlas/raw_data/agolini/20201218_LZH-
AG_MdR_ENIGMA_Atlas_20201111rnd1_QE119_Ag683775-924_USHXG01474/2020121
8_LZH-
AG_MdR_ENIGMA_Atlas_20201111rnd1_QE119_Ag683775-924_USHXG01474_POS_MSM
S-v2_26_NLDM-AMD892-pl2_2__160.mzML</file><file>/global/project/
projectdirs/metatlas/raw_data/agolini/20201218_LZH-
AG_MdR_ENIGMA_Atlas_20201111rnd1_QE119_Ag683775-924_USHXG01474/2020121
8_LZH-
AG_MdR_ENIGMA_Atlas_20201111rnd1_QE119_Ag683775-924_USHXG01474_POS_MSM
S-v2_27_NLDM-AMD892-pl2_3__121.mzML</file><file>/global/project/
projectdirs/metatlas/raw_data/agolini/20201218_LZH-
AG_MdR_ENIGMA_Atlas_20201111rnd1_QE119_Ag683775-924_USHXG01474/2020121
8_LZH-
AG_MdR_ENIGMA_Atlas_20201111rnd1_QE119_Ag683775-924_USHXG01474_POS_MSM
S-v2_28_NLDM-AMD66-pl2_1__058.mzML</file><file>/global/project/
projectdirs/metatlas/raw_data/agolini/20201218_LZH-
AG_MdR_ENIGMA_Atlas_20201111rnd1_QE119_Ag683775-924_USHXG01474/2020121
8_LZH-
AG_MdR_ENIGMA_Atlas_20201111rnd1_QE119_Ag683775-924_USHXG01474_POS_MSM
S-v2_29_NLDM-AMD66-pl2_2__276.mzML</file><file>/global/project/

projectdirs/metatlas/raw_data/agolini/20201218_LZH-
AG_MdR_ENIGMA_Atlas_20201111rnd1_QE119_Ag683775-924_USHXG01474/2020121
8_LZH-
AG_MdR_ENIGMA_Atlas_20201111rnd1_QE119_Ag683775-924_USHXG01474_POS_MSM
S-v2_2_NLDM-AMD35-pl1_2__325.mzML</file><file>/global/project/
projectdirs/metatlas/raw_data/agolini/20201218_LZH-
AG_MdR_ENIGMA_Atlas_20201111rnd1_QE119_Ag683775-924_USHXG01474/2020121
8_LZH-
AG_MdR_ENIGMA_Atlas_20201111rnd1_QE119_Ag683775-924_USHXG01474_POS_MSM
S-v2_30_NLDM-AMD66-pl2_3__244.mzML</file><file>/global/project/
projectdirs/metatlas/raw_data/agolini/20201218_LZH-
AG_MdR_ENIGMA_Atlas_20201111rnd1_QE119_Ag683775-924_USHXG01474/2020121
8_LZH-
AG_MdR_ENIGMA_Atlas_20201111rnd1_QE119_Ag683775-924_USHXG01474_POS_MSM
S-v2_31_NLDM-AMD171-pl2_1__340.mzML</file><file>/global/project/
projectdirs/metatlas/raw_data/agolini/20201218_LZH-
AG_MdR_ENIGMA_Atlas_20201111rnd1_QE119_Ag683775-924_USHXG01474/2020121
8_LZH-
AG_MdR_ENIGMA_Atlas_20201111rnd1_QE119_Ag683775-924_USHXG01474_POS_MSM
S-v2_32_NLDM-AMD171-pl2_2__376.mzML</file><file>/global/project/
projectdirs/metatlas/raw_data/agolini/20201218_LZH-
AG_MdR_ENIGMA_Atlas_20201111rnd1_QE119_Ag683775-924_USHXG01474/2020121
8_LZH-
AG_MdR_ENIGMA_Atlas_20201111rnd1_QE119_Ag683775-924_USHXG01474_POS_MSM
S-v2_33_NLDM-AMD171-pl2_3__385.mzML</file><file>/global/project/
projectdirs/metatlas/raw_data/agolini/20201218_LZH-
AG_MdR_ENIGMA_Atlas_20201111rnd1_QE119_Ag683775-924_USHXG01474/2020121
8_LZH-
AG_MdR_ENIGMA_Atlas_20201111rnd1_QE119_Ag683775-924_USHXG01474_POS_MSM
S-v2_34_NLDM-AMD174-pl2_1__064.mzML</file><file>/global/project/
projectdirs/metatlas/raw_data/agolini/20201218_LZH-
AG_MdR_ENIGMA_Atlas_20201111rnd1_QE119_Ag683775-924_USHXG01474/2020121
8_LZH-
AG_MdR_ENIGMA_Atlas_20201111rnd1_QE119_Ag683775-924_USHXG01474_POS_MSM
S-v2_35_NLDM-AMD174-pl2_2__232.mzML</file><file>/global/project/
projectdirs/metatlas/raw_data/agolini/20201218_LZH-
AG_MdR_ENIGMA_Atlas_20201111rnd1_QE119_Ag683775-924_USHXG01474/2020121
8_LZH-
AG_MdR_ENIGMA_Atlas_20201111rnd1_QE119_Ag683775-924_USHXG01474_POS_MSM
S-v2_36_NLDM-AMD174-pl2_3__073.mzML</file><file>/global/project/
projectdirs/metatlas/raw_data/agolini/20201218_LZH-
AG_MdR_ENIGMA_Atlas_20201111rnd1_QE119_Ag683775-924_USHXG01474/2020121
8_LZH-
AG_MdR_ENIGMA_Atlas_20201111rnd1_QE119_Ag683775-924_USHXG01474_POS_MSM
S-v2_37_NLDM-AMD437-pl2_1__301.mzML</file><file>/global/project/
projectdirs/metatlas/raw_data/agolini/20201218_LZH-
AG_MdR_ENIGMA_Atlas_20201111rnd1_QE119_Ag683775-924_USHXG01474/2020121
8_LZH-
AG_MdR_ENIGMA_Atlas_20201111rnd1_QE119_Ag683775-924_USHXG01474_POS_MSM
S-v2_38_NLDM-AMD437-pl2_2__319.mzML</file><file>/global/project/

projectdirs/metatlas/raw_data/agolini/20201218_LZH-
AG_MdR_ENIGMA_Atlas_20201111rnd1_QE119_Ag683775-924_USHXG01474/2020121
8_LZH-
AG_MdR_ENIGMA_Atlas_20201111rnd1_QE119_Ag683775-924_USHXG01474_POS_MSM
S-v2_39_NLDM-AMD437-pl2_3__304.mzML</file><file>/global/project/
projectdirs/metatlas/raw_data/agolini/20201218_LZH-
AG_MdR_ENIGMA_Atlas_20201111rnd1_QE119_Ag683775-924_USHXG01474/2020121
8_LZH-
AG_MdR_ENIGMA_Atlas_20201111rnd1_QE119_Ag683775-924_USHXG01474_POS_MSM
S-v2_3_NLDM-AMD35-pl1_3__028.mzML</file><file>/global/project/
projectdirs/metatlas/raw_data/agolini/20201218_LZH-
AG_MdR_ENIGMA_Atlas_20201111rnd1_QE119_Ag683775-924_USHXG01474/2020121
8_LZH-
AG_MdR_ENIGMA_Atlas_20201111rnd1_QE119_Ag683775-924_USHXG01474_POS_MSM
S-v2_40_NLDM-AMD438-pl2_1__310.mzML</file><file>/global/project/
projectdirs/metatlas/raw_data/agolini/20201218_LZH-
AG_MdR_ENIGMA_Atlas_20201111rnd1_QE119_Ag683775-924_USHXG01474/2020121
8_LZH-
AG_MdR_ENIGMA_Atlas_20201111rnd1_QE119_Ag683775-924_USHXG01474_POS_MSM
S-v2_41_NLDM-AMD438-pl2_2__040.mzML</file><file>/global/project/
projectdirs/metatlas/raw_data/agolini/20201218_LZH-
AG_MdR_ENIGMA_Atlas_20201111rnd1_QE119_Ag683775-924_USHXG01474/2020121
8_LZH-
AG_MdR_ENIGMA_Atlas_20201111rnd1_QE119_Ag683775-924_USHXG01474_POS_MSM
S-v2_42_NLDM-AMD438-pl2_3__331.mzML</file><file>/global/project/
projectdirs/metatlas/raw_data/agolini/20201218_LZH-
AG_MdR_ENIGMA_Atlas_20201111rnd1_QE119_Ag683775-924_USHXG01474/2020121
8_LZH-
AG_MdR_ENIGMA_Atlas_20201111rnd1_QE119_Ag683775-924_USHXG01474_POS_MSM
S-v2_43_NLDM-AMD439-pl2_1__211.mzML</file><file>/global/project/
projectdirs/metatlas/raw_data/agolini/20201218_LZH-
AG_MdR_ENIGMA_Atlas_20201111rnd1_QE119_Ag683775-924_USHXG01474/2020121
8_LZH-
AG_MdR_ENIGMA_Atlas_20201111rnd1_QE119_Ag683775-924_USHXG01474_POS_MSM
S-v2_44_NLDM-AMD439-pl2_2__273.mzML</file><file>/global/project/
projectdirs/metatlas/raw_data/agolini/20201218_LZH-
AG_MdR_ENIGMA_Atlas_20201111rnd1_QE119_Ag683775-924_USHXG01474/2020121
8_LZH-
AG_MdR_ENIGMA_Atlas_20201111rnd1_QE119_Ag683775-924_USHXG01474_POS_MSM
S-v2_45_NLDM-AMD439-pl2_3__196.mzML</file><file>/global/project/
projectdirs/metatlas/raw_data/agolini/20201218_LZH-
AG_MdR_ENIGMA_Atlas_20201111rnd1_QE119_Ag683775-924_USHXG01474/2020121
8_LZH-
AG_MdR_ENIGMA_Atlas_20201111rnd1_QE119_Ag683775-924_USHXG01474_POS_MSM
S-v2_46_NLDM-TxCtrl-pl2_1__139.mzML</file><file>/global/project/
projectdirs/metatlas/raw_data/agolini/20201218_LZH-
AG_MdR_ENIGMA_Atlas_20201111rnd1_QE119_Ag683775-924_USHXG01474/2020121
8_LZH-
AG_MdR_ENIGMA_Atlas_20201111rnd1_QE119_Ag683775-924_USHXG01474_POS_MSM
S-v2_47_NLDM-TxCtrl-pl2_2__142.mzML</file><file>/global/project/

projectdirs/metatlas/raw_data/agolini/20201218_LZH-
AG_MdR_ENIGMA_Atlas_20201111rnd1_QE119_Ag683775-924_USHXG01474/2020121
8_LZH-
AG_MdR_ENIGMA_Atlas_20201111rnd1_QE119_Ag683775-924_USHXG01474_POS_MSM
S-v2_48_NLDM-TxCtrl-pl2_3__031.mzML</file><file>/global/project/
projectdirs/metatlas/raw_data/agolini/20201218_LZH-
AG_MdR_ENIGMA_Atlas_20201111rnd1_QE119_Ag683775-924_USHXG01474/2020121
8_LZH-
AG_MdR_ENIGMA_Atlas_20201111rnd1_QE119_Ag683775-924_USHXG01474_POS_MSM
S-v2_49_NLDM-AMD441-pl3_1__166.mzML</file><file>/global/project/
projectdirs/metatlas/raw_data/agolini/20201218_LZH-
AG_MdR_ENIGMA_Atlas_20201111rnd1_QE119_Ag683775-924_USHXG01474/2020121
8_LZH-
AG_MdR_ENIGMA_Atlas_20201111rnd1_QE119_Ag683775-924_USHXG01474_POS_MSM
S-v2_4_NLDM-AMD173-pl1_1__130.mzML</file><file>/global/project/
projectdirs/metatlas/raw_data/agolini/20201218_LZH-
AG_MdR_ENIGMA_Atlas_20201111rnd1_QE119_Ag683775-924_USHXG01474/2020121
8_LZH-
AG_MdR_ENIGMA_Atlas_20201111rnd1_QE119_Ag683775-924_USHXG01474_POS_MSM
S-v2_50_NLDM-AMD441-pl3_2__285.mzML</file><file>/global/project/
projectdirs/metatlas/raw_data/agolini/20201218_LZH-
AG_MdR_ENIGMA_Atlas_20201111rnd1_QE119_Ag683775-924_USHXG01474/2020121
8_LZH-
AG_MdR_ENIGMA_Atlas_20201111rnd1_QE119_Ag683775-924_USHXG01474_POS_MSM
S-v2_51_NLDM-AMD441-pl3_3__124.mzML</file><file>/global/project/
projectdirs/metatlas/raw_data/agolini/20201218_LZH-
AG_MdR_ENIGMA_Atlas_20201111rnd1_QE119_Ag683775-924_USHXG01474/2020121
8_LZH-
AG_MdR_ENIGMA_Atlas_20201111rnd1_QE119_Ag683775-924_USHXG01474_POS_MSM
S-v2_52_NLDM-AMD891-pl3_1__169.mzML</file><file>/global/project/
projectdirs/metatlas/raw_data/agolini/20201218_LZH-
AG_MdR_ENIGMA_Atlas_20201111rnd1_QE119_Ag683775-924_USHXG01474/2020121
8_LZH-
AG_MdR_ENIGMA_Atlas_20201111rnd1_QE119_Ag683775-924_USHXG01474_POS_MSM
S-v2_53_NLDM-AMD891-pl3_2__379.mzML</file><file>/global/project/
projectdirs/metatlas/raw_data/agolini/20201218_LZH-
AG_MdR_ENIGMA_Atlas_20201111rnd1_QE119_Ag683775-924_USHXG01474/2020121
8_LZH-
AG_MdR_ENIGMA_Atlas_20201111rnd1_QE119_Ag683775-924_USHXG01474_POS_MSM
S-v2_54_NLDM-AMD891-pl3_3__175.mzML</file><file>/global/project/
projectdirs/metatlas/raw_data/agolini/20201218_LZH-
AG_MdR_ENIGMA_Atlas_20201111rnd1_QE119_Ag683775-924_USHXG01474/2020121
8_LZH-
AG_MdR_ENIGMA_Atlas_20201111rnd1_QE119_Ag683775-924_USHXG01474_POS_MSM
S-v2_55_NLDM-TxCtrl-pl3_1__145.mzML</file><file>/global/project/
projectdirs/metatlas/raw_data/agolini/20201218_LZH-
AG_MdR_ENIGMA_Atlas_20201111rnd1_QE119_Ag683775-924_USHXG01474/2020121
8_LZH-
AG_MdR_ENIGMA_Atlas_20201111rnd1_QE119_Ag683775-924_USHXG01474_POS_MSM
S-v2_56_NLDM-TxCtrl-pl3_2__343.mzML</file><file>/global/project/

projectdirs/metatlas/raw_data/agolini/20201218_LZH-
AG_MdR_ENIGMA_Atlas_20201111rnd1_QE119_Ag683775-924_USHXG01474/2020121
8_LZH-
AG_MdR_ENIGMA_Atlas_20201111rnd1_QE119_Ag683775-924_USHXG01474_POS_MSM
S-v2_57_NLDM-TxCtrl-pl3_3__151.mzML</file><file>/global/project/
projectdirs/metatlas/raw_data/agolini/20201218_LZH-
AG_MdR_ENIGMA_Atlas_20201111rnd1_QE119_Ag683775-924_USHXG01474/2020121
8_LZH-
AG_MdR_ENIGMA_Atlas_20201111rnd1_QE119_Ag683775-924_USHXG01474_POS_MSM
S-v2_58_NLDM-AMD167-pl3_1__094.mzML</file><file>/global/project/
projectdirs/metatlas/raw_data/agolini/20201218_LZH-
AG_MdR_ENIGMA_Atlas_20201111rnd1_QE119_Ag683775-924_USHXG01474/2020121
8_LZH-
AG_MdR_ENIGMA_Atlas_20201111rnd1_QE119_Ag683775-924_USHXG01474_POS_MSM
S-v2_59_NLDM-AMD167-pl3_2__199.mzML</file><file>/global/project/
projectdirs/metatlas/raw_data/agolini/20201218_LZH-
AG_MdR_ENIGMA_Atlas_20201111rnd1_QE119_Ag683775-924_USHXG01474/2020121
8_LZH-
AG_MdR_ENIGMA_Atlas_20201111rnd1_QE119_Ag683775-924_USHXG01474_POS_MSM
S-v2_5_NLDM-AMD173-pl1_2__391.mzML</file><file>/global/project/
projectdirs/metatlas/raw_data/agolini/20201218_LZH-
AG_MdR_ENIGMA_Atlas_20201111rnd1_QE119_Ag683775-924_USHXG01474/2020121
8_LZH-
AG_MdR_ENIGMA_Atlas_20201111rnd1_QE119_Ag683775-924_USHXG01474_POS_MSM
S-v2_60_NLDM-AMD167-pl3_3__328.mzML</file><file>/global/project/
projectdirs/metatlas/raw_data/agolini/20201218_LZH-
AG_MdR_ENIGMA_Atlas_20201111rnd1_QE119_Ag683775-924_USHXG01474/2020121
8_LZH-
AG_MdR_ENIGMA_Atlas_20201111rnd1_QE119_Ag683775-924_USHXG01474_POS_MSM
S-v2_61_NLDM-AMD202-pl3_1__067.mzML</file><file>/global/project/
projectdirs/metatlas/raw_data/agolini/20201218_LZH-
AG_MdR_ENIGMA_Atlas_20201111rnd1_QE119_Ag683775-924_USHXG01474/2020121
8_LZH-
AG_MdR_ENIGMA_Atlas_20201111rnd1_QE119_Ag683775-924_USHXG01474_POS_MSM
S-v2_62_NLDM-AMD202-pl3_2__364.mzML</file><file>/global/project/
projectdirs/metatlas/raw_data/agolini/20201218_LZH-
AG_MdR_ENIGMA_Atlas_20201111rnd1_QE119_Ag683775-924_USHXG01474/2020121
8_LZH-
AG_MdR_ENIGMA_Atlas_20201111rnd1_QE119_Ag683775-924_USHXG01474_POS_MSM
S-v2_63_NLDM-AMD202-pl3_3__178.mzML</file><file>/global/project/
projectdirs/metatlas/raw_data/agolini/20201218_LZH-
AG_MdR_ENIGMA_Atlas_20201111rnd1_QE119_Ag683775-924_USHXG01474/2020121
8_LZH-
AG_MdR_ENIGMA_Atlas_20201111rnd1_QE119_Ag683775-924_USHXG01474_POS_MSM
S-v2_64_NLDM-AMD514-pl3_1__355.mzML</file><file>/global/project/
projectdirs/metatlas/raw_data/agolini/20201218_LZH-
AG_MdR_ENIGMA_Atlas_20201111rnd1_QE119_Ag683775-924_USHXG01474/2020121
8_LZH-
AG_MdR_ENIGMA_Atlas_20201111rnd1_QE119_Ag683775-924_USHXG01474_POS_MSM
S-v2_65_NLDM-AMD514-pl3_2__361.mzML</file><file>/global/project/

projectdirs/metatlas/raw_data/agolini/20201218_LZH-
AG_MdR_ENIGMA_Atlas_20201111rnd1_QE119_Ag683775-924_USHXG01474/2020121
8_LZH-
AG_MdR_ENIGMA_Atlas_20201111rnd1_QE119_Ag683775-924_USHXG01474_POS_MSM
S-v2_66_NLDM-AMD514-pl3_3__258.mzML</file><file>/global/project/
projectdirs/metatlas/raw_data/agolini/20201218_LZH-
AG_MdR_ENIGMA_Atlas_20201111rnd1_QE119_Ag683775-924_USHXG01474/2020121
8_LZH-
AG_MdR_ENIGMA_Atlas_20201111rnd1_QE119_Ag683775-924_USHXG01474_POS_MSM
S-v2_67_NLDM-AMD557-pl3_1__202.mzML</file><file>/global/project/
projectdirs/metatlas/raw_data/agolini/20201218_LZH-
AG_MdR_ENIGMA_Atlas_20201111rnd1_QE119_Ag683775-924_USHXG01474/2020121
8_LZH-
AG_MdR_ENIGMA_Atlas_20201111rnd1_QE119_Ag683775-924_USHXG01474_POS_MSM
S-v2_68_NLDM-AMD557-pl3_2__103.mzML</file><file>/global/project/
projectdirs/metatlas/raw_data/agolini/20201218_LZH-
AG_MdR_ENIGMA_Atlas_20201111rnd1_QE119_Ag683775-924_USHXG01474/2020121
8_LZH-
AG_MdR_ENIGMA_Atlas_20201111rnd1_QE119_Ag683775-924_USHXG01474_POS_MSM
S-v2_69_NLDM-AMD557-pl3_3__034.mzML</file><file>/global/project/
projectdirs/metatlas/raw_data/agolini/20201218_LZH-
AG_MdR_ENIGMA_Atlas_20201111rnd1_QE119_Ag683775-924_USHXG01474/2020121
8_LZH-
AG_MdR_ENIGMA_Atlas_20201111rnd1_QE119_Ag683775-924_USHXG01474_POS_MSM
S-v2_6_NLDM-AMD173-pl1_3__133.mzML</file><file>/global/project/
projectdirs/metatlas/raw_data/agolini/20201218_LZH-
AG_MdR_ENIGMA_Atlas_20201111rnd1_QE119_Ag683775-924_USHXG01474/2020121
8_LZH-
AG_MdR_ENIGMA_Atlas_20201111rnd1_QE119_Ag683775-924_USHXG01474_POS_MSM
S-v2_70_NLDM-TxCtrl-pl3_4__157.mzML</file><file>/global/project/
projectdirs/metatlas/raw_data/agolini/20201218_LZH-
AG_MdR_ENIGMA_Atlas_20201111rnd1_QE119_Ag683775-924_USHXG01474/2020121
8_LZH-
AG_MdR_ENIGMA_Atlas_20201111rnd1_QE119_Ag683775-924_USHXG01474_POS_MSM
S-v2_71_NLDM-TxCtrl-pl3_5__184.mzML</file><file>/global/project/
projectdirs/metatlas/raw_data/agolini/20201218_LZH-
AG_MdR_ENIGMA_Atlas_20201111rnd1_QE119_Ag683775-924_USHXG01474/2020121
8_LZH-
AG_MdR_ENIGMA_Atlas_20201111rnd1_QE119_Ag683775-924_USHXG01474_POS_MSM
S-v2_72_NLDM-TxCtrl-pl3_6__279.mzML</file><file>/global/project/
projectdirs/metatlas/raw_data/agolini/20201218_LZH-
AG_MdR_ENIGMA_Atlas_20201111rnd1_QE119_Ag683775-924_USHXG01474/2020121
8_LZH-
AG_MdR_ENIGMA_Atlas_20201111rnd1_QE119_Ag683775-924_USHXG01474_POS_MSM
S-v2_73_NLDM-FHT5-A05-pl4_1__235.mzML</file><file>/global/project/
projectdirs/metatlas/raw_data/agolini/20201218_LZH-
AG_MdR_ENIGMA_Atlas_20201111rnd1_QE119_Ag683775-924_USHXG01474/2020121
8_LZH-
AG_MdR_ENIGMA_Atlas_20201111rnd1_QE119_Ag683775-924_USHXG01474_POS_MSM
S-v2_74_NLDM-FHT5-A05-pl4_2__270.mzML</file><file>/global/project/

projectdirs/metatlas/raw_data/agolini/20201218_LZH-
AG_MdR_ENIGMA_Atlas_20201111rnd1_QE119_Ag683775-924_USHXG01474/2020121
8_LZH-
AG_MdR_ENIGMA_Atlas_20201111rnd1_QE119_Ag683775-924_USHXG01474_POS_MSM
S-v2_75_NLDM-FHT5-A05-pl4_3__070.mzML</file><file>/global/project/
projectdirs/metatlas/raw_data/agolini/20201218_LZH-
AG_MdR_ENIGMA_Atlas_20201111rnd1_QE119_Ag683775-924_USHXG01474/2020121
8_LZH-
AG_MdR_ENIGMA_Atlas_20201111rnd1_QE119_Ag683775-924_USHXG01474_POS_MSM
S-v2_76_NLDM-FHT5-B04-pl4_1__255.mzML</file><file>/global/project/
projectdirs/metatlas/raw_data/agolini/20201218_LZH-
AG_MdR_ENIGMA_Atlas_20201111rnd1_QE119_Ag683775-924_USHXG01474/2020121
8_LZH-
AG_MdR_ENIGMA_Atlas_20201111rnd1_QE119_Ag683775-924_USHXG01474_POS_MSM
S-v2_77_NLDM-FHT5-B04-pl4_2__076.mzML</file><file>/global/project/
projectdirs/metatlas/raw_data/agolini/20201218_LZH-
AG_MdR_ENIGMA_Atlas_20201111rnd1_QE119_Ag683775-924_USHXG01474/2020121
8_LZH-
AG_MdR_ENIGMA_Atlas_20201111rnd1_QE119_Ag683775-924_USHXG01474_POS_MSM
S-v2_78_NLDM-FHT5-B04-pl4_3__190.mzML</file><file>/global/project/
projectdirs/metatlas/raw_data/agolini/20201218_LZH-
AG_MdR_ENIGMA_Atlas_20201111rnd1_QE119_Ag683775-924_USHXG01474/2020121
8_LZH-
AG_MdR_ENIGMA_Atlas_20201111rnd1_QE119_Ag683775-924_USHXG01474_POS_MSM
S-v2_79_NLDM-FHT5-B06-pl4_1__352.mzML</file><file>/global/project/
projectdirs/metatlas/raw_data/agolini/20201218_LZH-
AG_MdR_ENIGMA_Atlas_20201111rnd1_QE119_Ag683775-924_USHXG01474/2020121
8_LZH-
AG_MdR_ENIGMA_Atlas_20201111rnd1_QE119_Ag683775-924_USHXG01474_POS_MSM
S-v2_7_NLDM-AMD436-pl1_1__046.mzML</file><file>/global/project/
projectdirs/metatlas/raw_data/agolini/20201218_LZH-
AG_MdR_ENIGMA_Atlas_20201111rnd1_QE119_Ag683775-924_USHXG01474/2020121
8_LZH-
AG_MdR_ENIGMA_Atlas_20201111rnd1_QE119_Ag683775-924_USHXG01474_POS_MSM
S-v2_80_NLDM-FHT5-B06-pl4_2__294.mzML</file><file>/global/project/
projectdirs/metatlas/raw_data/agolini/20201218_LZH-
AG_MdR_ENIGMA_Atlas_20201111rnd1_QE119_Ag683775-924_USHXG01474/2020121
8_LZH-
AG_MdR_ENIGMA_Atlas_20201111rnd1_QE119_Ag683775-924_USHXG01474_POS_MSM
S-v2_81_NLDM-FHT5-B06-pl4_3__367.mzML</file><file>/global/project/
projectdirs/metatlas/raw_data/agolini/20201218_LZH-
AG_MdR_ENIGMA_Atlas_20201111rnd1_QE119_Ag683775-924_USHXG01474/2020121
8_LZH-
AG_MdR_ENIGMA_Atlas_20201111rnd1_QE119_Ag683775-924_USHXG01474_POS_MSM
S-v2_82_NLDM-FHT5-B07-pl4_1__127.mzML</file><file>/global/project/
projectdirs/metatlas/raw_data/agolini/20201218_LZH-
AG_MdR_ENIGMA_Atlas_20201111rnd1_QE119_Ag683775-924_USHXG01474/2020121
8_LZH-
AG_MdR_ENIGMA_Atlas_20201111rnd1_QE119_Ag683775-924_USHXG01474_POS_MSM
S-v2_83_NLDM-FHT5-B07-pl4_2__223.mzML</file><file>/global/project/

projectdirs/metatlas/raw_data/agolini/20201218_LZH-
AG_MdR_ENIGMA_Atlas_20201111rnd1_QE119_Ag683775-924_USHXG01474/2020121
8_LZH-
AG_MdR_ENIGMA_Atlas_20201111rnd1_QE119_Ag683775-924_USHXG01474_POS_MSM
S-v2_84_NLDM-FHT5-B07-pl4_3__334.mzML</file><file>/global/project/
projectdirs/metatlas/raw_data/agolini/20201218_LZH-
AG_MdR_ENIGMA_Atlas_20201111rnd1_QE119_Ag683775-924_USHXG01474/2020121
8_LZH-
AG_MdR_ENIGMA_Atlas_20201111rnd1_QE119_Ag683775-924_USHXG01474_POS_MSM
S-v2_85_NLDM-FHT5-C12-pl4_1__163.mzML</file><file>/global/project/
projectdirs/metatlas/raw_data/agolini/20201218_LZH-
AG_MdR_ENIGMA_Atlas_20201111rnd1_QE119_Ag683775-924_USHXG01474/2020121
8_LZH-
AG_MdR_ENIGMA_Atlas_20201111rnd1_QE119_Ag683775-924_USHXG01474_POS_MSM
S-v2_86_NLDM-FHT5-C12-pl4_2__052.mzML</file><file>/global/project/
projectdirs/metatlas/raw_data/agolini/20201218_LZH-
AG_MdR_ENIGMA_Atlas_20201111rnd1_QE119_Ag683775-924_USHXG01474/2020121
8_LZH-
AG_MdR_ENIGMA_Atlas_20201111rnd1_QE119_Ag683775-924_USHXG01474_POS_MSM
S-v2_87_NLDM-FHT5-C12-pl4_3__193.mzML</file><file>/global/project/
projectdirs/metatlas/raw_data/agolini/20201218_LZH-
AG_MdR_ENIGMA_Atlas_20201111rnd1_QE119_Ag683775-924_USHXG01474/2020121
8_LZH-
AG_MdR_ENIGMA_Atlas_20201111rnd1_QE119_Ag683775-924_USHXG01474_POS_MSM
S-v2_88_NLDM-FHT5-D05-pl4_1__079.mzML</file><file>/global/project/
projectdirs/metatlas/raw_data/agolini/20201218_LZH-
AG_MdR_ENIGMA_Atlas_20201111rnd1_QE119_Ag683775-924_USHXG01474/2020121
8_LZH-
AG_MdR_ENIGMA_Atlas_20201111rnd1_QE119_Ag683775-924_USHXG01474_POS_MSM
S-v2_89_NLDM-FHT5-D05-pl4_2__241.mzML</file><file>/global/project/
projectdirs/metatlas/raw_data/agolini/20201218_LZH-
AG_MdR_ENIGMA_Atlas_20201111rnd1_QE119_Ag683775-924_USHXG01474/2020121
8_LZH-
AG_MdR_ENIGMA_Atlas_20201111rnd1_QE119_Ag683775-924_USHXG01474_POS_MSM
S-v2_8_NLDM-AMD436-pl1_2__055.mzML</file><file>/global/project/
projectdirs/metatlas/raw_data/agolini/20201218_LZH-
AG_MdR_ENIGMA_Atlas_20201111rnd1_QE119_Ag683775-924_USHXG01474/2020121
8_LZH-
AG_MdR_ENIGMA_Atlas_20201111rnd1_QE119_Ag683775-924_USHXG01474_POS_MSM
S-v2_90_NLDM-FHT5-D05-pl4_3__226.mzML</file><file>/global/project/
projectdirs/metatlas/raw_data/agolini/20201218_LZH-
AG_MdR_ENIGMA_Atlas_20201111rnd1_QE119_Ag683775-924_USHXG01474/2020121
8_LZH-
AG_MdR_ENIGMA_Atlas_20201111rnd1_QE119_Ag683775-924_USHXG01474_POS_MSM
S-v2_91_NLDM-FHT5-D12-pl4_1__358.mzML</file><file>/global/project/
projectdirs/metatlas/raw_data/agolini/20201218_LZH-
AG_MdR_ENIGMA_Atlas_20201111rnd1_QE119_Ag683775-924_USHXG01474/2020121
8_LZH-
AG_MdR_ENIGMA_Atlas_20201111rnd1_QE119_Ag683775-924_USHXG01474_POS_MSM
S-v2_92_NLDM-FHT5-D12-pl4_2__313.mzML</file><file>/global/project/

projectdirs/metatlas/raw_data/agolini/20201218_LZH-
AG_MdR_ENIGMA_Atlas_20201111rnd1_QE119_Ag683775-924_USHXG01474/2020121
8_LZH-
AG_MdR_ENIGMA_Atlas_20201111rnd1_QE119_Ag683775-924_USHXG01474_POS_MSM
S-v2_93_NLDM-FHT5-D12-pl4_3_failedinj-2mm-noWBS_019.mzML</file><file>/
global/project/projectdirs/metatlas/raw_data/agolini/20201218_LZH-
AG_MdR_ENIGMA_Atlas_20201111rnd1_QE119_Ag683775-924_USHXG01474/2020121
8_LZH-
AG_MdR_ENIGMA_Atlas_20201111rnd1_QE119_Ag683775-924_USHXG01474_POS_MSM
S-v2_93_NLDM-FHT5-D12-pl4_3_reinj-postfailed_397.mzML</file><file>/
global/project/projectdirs/metatlas/raw_data/agolini/20201218_LZH-
AG_MdR_ENIGMA_Atlas_20201111rnd1_QE119_Ag683775-924_USHXG01474/2020121
8_LZH-
AG_MdR_ENIGMA_Atlas_20201111rnd1_QE119_Ag683775-924_USHXG01474_POS_MSM
S-v2_94_NLDM-TxCtrl-pl4_1__322.mzML</file><file>/global/project/
projectdirs/metatlas/raw_data/agolini/20201218_LZH-
AG_MdR_ENIGMA_Atlas_20201111rnd1_QE119_Ag683775-924_USHXG01474/2020121
8_LZH-
AG_MdR_ENIGMA_Atlas_20201111rnd1_QE119_Ag683775-924_USHXG01474_POS_MSM
S-v2_95_NLDM-TxCtrl-pl4_2__264.mzML</file><file>/global/project/
projectdirs/metatlas/raw_data/agolini/20201218_LZH-
AG_MdR_ENIGMA_Atlas_20201111rnd1_QE119_Ag683775-924_USHXG01474/2020121
8_LZH-
AG_MdR_ENIGMA_Atlas_20201111rnd1_QE119_Ag683775-924_USHXG01474_POS_MSM
S-v2_96_NLDM-TxCtrl-pl4_3__217.mzML</file><file>/global/project/
projectdirs/metatlas/raw_data/agolini/20201218_LZH-
AG_MdR_ENIGMA_Atlas_20201111rnd1_QE119_Ag683775-924_USHXG01474/2020121
8_LZH-
AG_MdR_ENIGMA_Atlas_20201111rnd1_QE119_Ag683775-924_USHXG01474_POS_MSM
S-v2_97_NLDM-FHT5-F08-pl5_1__187.mzML</file><file>/global/project/
projectdirs/metatlas/raw_data/agolini/20201218_LZH-
AG_MdR_ENIGMA_Atlas_20201111rnd1_QE119_Ag683775-924_USHXG01474/2020121
8_LZH-
AG_MdR_ENIGMA_Atlas_20201111rnd1_QE119_Ag683775-924_USHXG01474_POS_MSM
S-v2_98_NLDM-FHT5-F08-pl5_2__252.mzML</file><file>/global/project/
projectdirs/metatlas/raw_data/agolini/20201218_LZH-
AG_MdR_ENIGMA_Atlas_20201111rnd1_QE119_Ag683775-924_USHXG01474/2020121
8_LZH-
AG_MdR_ENIGMA_Atlas_20201111rnd1_QE119_Ag683775-924_USHXG01474_POS_MSM
S-v2_99_NLDM-FHT5-F08-pl5_3__346.mzML</file><file>/global/project/
projectdirs/metatlas/raw_data/agolini/20201218_LZH-
AG_MdR_ENIGMA_Atlas_20201111rnd1_QE119_Ag683775-924_USHXG01474/2020121
8_LZH-
AG_MdR_ENIGMA_Atlas_20201111rnd1_QE119_Ag683775-924_USHXG01474_POS_MSM
S-v2_9_NLDM-AMD436-pl1_3__373.mzML</file></parameter></
batchstep><batchstep
method="net.sf.mzmine.modules.rawdatamethods.peakpicking.massdetection
.MassDetectionModule"><parameter name="Raw data files"
type="ALL_FILES"></parameter><parameter name="Scans"><ms_level>1</
ms_level><scan_definition></scan_definition></parameter><parameter

```
name="Mass detector" selected="Centroid"><module
name="Centroid"><parameter name="Noise level">10000</parameter></
module><module name="Exact mass"><parameter name="Noise level"></
parameter></module><module name="Local maxima"><parameter name="Noise
level"></parameter></module><module name="Recursive
threshold"><parameter name="Noise level"></parameter><parameter
name="Min m/z peak width"></parameter><parameter name="Max m/z peak
width"></parameter></module><module name="Wavelet
transform"><parameter name="Noise level"></parameter><parameter
name="Scale level"></parameter><parameter name="Wavelet window size
(%)"></parameter></module></parameter><parameter name="Mass list
name">masses</parameter><parameter name="CDF Filename (optional)"
selected="False"></parameter></batchstep><batchstep
method="net.sf.mzmine.modules.rawdatamethods.peakpicking.massdetection
.MassDetectionModule"><parameter name="Raw data files"
type="ALL_FILES"></parameter><parameter name="Scans"><ms_level>2</
ms_level><scan_definition></scan_definition></parameter><parameter
name="Mass detector" selected="Centroid"><module
name="Centroid"><parameter name="Noise level">100</parameter></
module><module name="Exact mass"><parameter name="Noise level"></
parameter></module><module name="Local maxima"><parameter name="Noise
level"></parameter></module><module name="Recursive
threshold"><parameter name="Noise level"></parameter><parameter
name="Min m/z peak width"></parameter><parameter name="Max m/z peak
width"></parameter></module><module name="Wavelet
transform"><parameter name="Noise level"></parameter><parameter
name="Scale level"></parameter><parameter name="Wavelet window size
(%)"></parameter></module></parameter><parameter name="Mass list
name">masses</parameter><parameter name="CDF Filename (optional)"
selected="False"></parameter></batchstep><batchstep
method="net.sf.mzmine.modules.masslistmethods.ADAPchromatogrambuilder.
ADAPChromatogramBuilderModule"><parameter name="Raw data files"
type="ALL_FILES"></parameter><parameter name="Scans"><ms_level>1</
ms_level></parameter><parameter name="Mass list">masses</
parameter><parameter name="Min group size in # of scans">5</
parameter><parameter name="Group intensity threshold">200000.0</
parameter><parameter name="Min highest intensity">500000.0</
parameter><parameter name="m/z tolerance"><absolutetolerance>0.002</
absolutetolerance><ppmtolerance>10</ppmtolerance></
parameter><parameter name="Suffix">chromatograms</parameter></
batchstep><batchstep
method="net.sf.mzmine.modules.peaklistmethods.peakpicking.deconvolutio
n.DeconvolutionModule"><parameter name="Peak lists"
type="BATCH_LAST_PEAKLISTS"></parameter><parameter
name="Suffix">deconvoluted</parameter><parameter name="Algorithm"
selected="Wavelets (ADAP)"><module name="Baseline cut-off"><parameter
name="Min peak height"></parameter><parameter name="Peak duration
range (min)"><max>10</max><min>0</min></parameter><parameter
name="Baseline level"></parameter></module><module name="Noise
amplitude"><parameter name="Min peak height"></parameter><parameter
```

name="Peak duration range (min)"><max>10</max><min>0</min></
parameter><parameter name="Amplitude of noise"></parameter></
module><module name="Savitzky-Golay"><parameter name="Min peak
height"></parameter><parameter name="Peak duration range
(min)"><max>10</max><min>0</min></parameter><parameter
name="Derivative threshold level"></parameter></module><module
name="Local minimum search"><parameter name="Chromatographic
threshold">0.3</parameter><parameter name="Search minimum in RT range
(min)">0.05</parameter><parameter name="Minimum relative
height">0.05</parameter><parameter name="Minimum absolute
height">1000000</parameter><parameter name="Min ratio of peak top/
edge">2</parameter><parameter name="Peak duration range
(min)"><max>2</max><min>0.05</min></parameter></module><module
name="Wavelets (XCMS)"><parameter name="S/N threshold">10</
parameter><parameter name="Wavelet scales"><max>5</max><min>0.25</
min></parameter><parameter name="Peak duration range"><max>10</
max><min>0</min></parameter><parameter name="Peak integration
method">Use smoothed data</parameter><parameter name="R
engine">RCaller</parameter></module><module name="Wavelets
(ADAP)"><parameter name="S/N threshold">10</parameter><parameter
name="S/N estimator" selected="Intensity window SN"><module
name="Intensity window SN"></module><module name="Wavelet Coeff.
SN"><parameter name="Peak width mult.">3</parameter><parameter
name="abs(wavelet coeffs.)">True</parameter></module></
parameter><parameter name="min feature height">10</
parameter><parameter name="coefficient/area threshold">110</
parameter><parameter name="Peak duration range"><max>10</max><min>0</
min></parameter><parameter name="RT wavelet range"><max>0.2</
max><min>0</min></parameter></module></parameter><parameter
measure="MEDIAN" name="m/z center calculation"
weighting="NONE">CenterFunction</parameter><parameter name="m/z range
for MS2 scan pairing (Da)" selected="True">0.025</parameter><parameter
name="RT range for MS2 scan pairing (min)" selected="True">0.2</
parameter><parameter name="Remove original peak list">False</
parameter></batchstep><batchstep
method="net.sf.mzmine.modules.peaklistmethods.isotopes.deisotoper.Isot
opeGrouperModule"><parameter name="Peak lists"
type="BATCH_LAST_PEAKLISTS"></parameter><parameter name="Name
suffix">deisotoped</parameter><parameter name="m/z
tolerance"><absolutetolerance>0</absolutetolerance><ppmtolerance>20</
ppmtolerance></parameter><parameter name="Retention time tolerance"
type="absolute">0.1</parameter><parameter name="Monotonic
shape">True</parameter><parameter name="Maximum charge">3</
parameter><parameter name="Representative isotope">Most intense</
parameter><parameter name="Remove original peaklist">True</
parameter></batchstep><batchstep
method="net.sf.mzmine.modules.peaklistmethods.orderpeaklists.OrderPeak
ListsModule"><parameter name="Peak lists"
type="BATCH_LAST_PEAKLISTS"></parameter></batchstep><batchstep
method="net.sf.mzmine.modules.peaklistmethods.alignment.join.JoinAlign

erModule"><parameter name="Peak lists" type="BATCH_LAST_PEAKLISTS"></
parameter><parameter name="Peak list name">Aligned peak list</
parameter><parameter name="m/z tolerance"><absolutetolerance>0.0</
absolutetolerance><ppmtolerance>20</ppmtolerance></
parameter><parameter name="Weight for m/z">50</parameter><parameter
name="Retention time tolerance" type="absolute">0.2</
parameter><parameter name="Weight for RT">50</parameter><parameter
name="Require same charge state">False</parameter><parameter
name="Require same ID">False</parameter><parameter name="Compare
isotope pattern" selected="False"><parameter name="Isotope m/z
tolerance"><absolutetolerance>0.001</
absolutetolerance><ppmtolerance>20</ppmtolerance></
parameter><parameter name="Minimum absolute intensity"></
parameter><parameter name="Minimum score"></parameter></parameter></
batchstep><batchstep
method="net.sf.mzmine.modules.peaklistmethods.filtering.rowsfilter.Row
sFilterModule"><parameter name="Peak lists"
type="BATCH_LAST_PEAKLISTS"></parameter><parameter name="Name
suffix">filtered</parameter><parameter name="Minimum peaks in a row"
selected="True">2</parameter><parameter name="Minimum peaks in an
isotope pattern" selected="True">2</parameter><parameter name="m/z"
selected="False"></parameter><parameter name="Retention time"
selected="False"></parameter><parameter name="Peak duration range"
selected="False"><max>10</max><min>0</min></parameter><parameter
name="Chromatographic FWHM" selected="False"><max>1</max><min>0</
min></parameter><parameter name="Parameter">No parameters defined</
parameter><parameter name="Only identified?">False</
parameter><parameter name="Text in identity" selected="False"></
parameter><parameter name="Text in comment" selected="False"></
parameter><parameter name="Keep or remove rows">Keep rows that match
all criteria</parameter><parameter name="Keep only peaks with MS2 scan
(GNPS)">False</parameter><parameter name="Reset the peak number
ID">False</parameter><parameter name="Remove source peak list after
filtering">False</parameter></batchstep><batchstep
method="net.sf.mzmine.modules.peaklistmethods.filtering.duplicatefilte
r.DuplicateFilterModule"><parameter name="Peak lists"
type="BATCH_LAST_PEAKLISTS"></parameter><parameter name="Name
suffix">filtered</parameter><parameter name="Filter mode">NEW
AVERAGE</parameter><parameter name="m/z
tolerance"><absolutetolerance>0.0001</
absolutetolerance><ppmtolerance>10</ppmtolerance></
parameter><parameter name="RT tolerance" type="absolute">0.05</
parameter><parameter name="Require same identification">False</
parameter><parameter name="Remove original peaklist">False</
parameter></batchstep><batchstep
method="net.sf.mzmine.modules.peaklistmethods.gapfilling.peakfinder.mu
ltithreaded.MultiThreadPeakFinderModule"><parameter name="Peak lists"
type="BATCH_LAST_PEAKLISTS"></parameter><parameter name="Name
suffix">gap-filled</parameter><parameter name="Intensity
tolerance">0.05</parameter><parameter name="m/z

tolerance"><absolutetolerance>0.001</
absolutetolerance><ppmtolerance>10</ppmtolerance></
parameter><parameter name="Retention time tolerance"
type="absolute">0.2</parameter><parameter name="Remove original peak
list">False</parameter></batchstep><batchstep
method="net.sf.mzmine.modules.peaklistmethods.io.csvexport.CSVExportMo
dule"><parameter name="Peak lists" type="BATCH_LAST_PEAKLISTS"></
parameter><parameter name="Filename">/project/projectdirs/metatlas/
projects/untargeted_tasks/20201218_LZH-
AG_MdR_ENIGMA_Atlas_20201111rnd1_QE119_Ag683775-924_USHXG01474_positiv
e/20201218_LZH-
AG_MdR_ENIGMA_Atlas_20201111rnd1_QE119_Ag683775-924_USHXG01474_positiv
e_peak-height.csv</parameter><parameter name="Field separator">,</
parameter><parameter name="Export common elements"><item>Export row
ID</item><item>Export row m/z</item><item>Export row retention time</
item></parameter><parameter name="Export data file
elements"><item>Peak height</item></parameter><parameter name="Export
quantitation results and other information">False</
parameter><parameter name="Identification separator">;</
parameter><parameter name="Filter rows">ALL</parameter></
batchstep><batchstep
method="net.sf.mzmine.modules.peaklistmethods.io.csvexport.CSVExportMo
dule"><parameter name="Peak lists" type="BATCH_LAST_PEAKLISTS"></
parameter><parameter name="Filename">/project/projectdirs/metatlas/
projects/untargeted_tasks/20201218_LZH-
AG_MdR_ENIGMA_Atlas_20201111rnd1_QE119_Ag683775-924_USHXG01474_positiv
e/20201218_LZH-
AG_MdR_ENIGMA_Atlas_20201111rnd1_QE119_Ag683775-924_USHXG01474_positiv
e_peak-area.csv</parameter><parameter name="Field separator">,</
parameter><parameter name="Export common elements"><item>Export row
ID</item><item>Export row m/z</item><item>Export row retention time</
item></parameter><parameter name="Export data file
elements"><item>Peak area</item></parameter><parameter name="Export
quantitation results and other information">False</
parameter><parameter name="Identification separator">;</
parameter><parameter name="Filter rows">ALL</parameter></
batchstep><batchstep
method="net.sf.mzmine.modules.peaklistmethods.io.gnpsexport.GNPSExport
AndSubmitModule"><parameter name="Peak lists"
type="BATCH_LAST_PEAKLISTS"></parameter><parameter name="Filename">/
project/projectdirs/metatlas/projects/untargeted_tasks/20201218_LZH-
AG_MdR_ENIGMA_Atlas_20201111rnd1_QE119_Ag683775-924_USHXG01474_positiv
e/20201218_LZH-
AG_MdR_ENIGMA_Atlas_20201111rnd1_QE119_Ag683775-924_USHXG01474_positiv
e_MSMS.mgf</parameter><parameter name="Mass list">masses</
parameter><parameter name="Filter rows">ONLY WITH MS2</
parameter><parameter name="Submit to GNPS" selected="False"><parameter
name="Meta data file" selected="True">/project/projectdirs/metatlas/
projects/untargeted_tasks/20201218_LZH-
AG_MdR_ENIGMA_Atlas_20201111rnd1_QE119_Ag683775-924_USHXG01474_positiv

e/20201218_LZH-
AG_MdR_ENIGMA_Atlas_20201111rnd1_QE119_Ag683775-924_USHXG01474_positiv
e_metadata.tab</parameter><parameter name="Presets">HIGHRES</
parameter><parameter name="Email">ben.bowen@gmail.com</
parameter><parameter name="Username">bpbowen</parameter><parameter
name="Password (unencrypted)">notapassword</parameter><parameter
name="Open website">False</parameter></parameter><parameter name="Open
folder">False</parameter></batchstep></batch>
