# Supplementary figures and images for: A Defined Medium for Cultivation and Exometabolite Profiling of Soil Bacteria
Source: Front Microbiol. 2022 May 25;13:855331. doi: 10.3389/fmicb.2022.855331 (PMC9174792; doi:10.3389/fmicb.2022.855331)

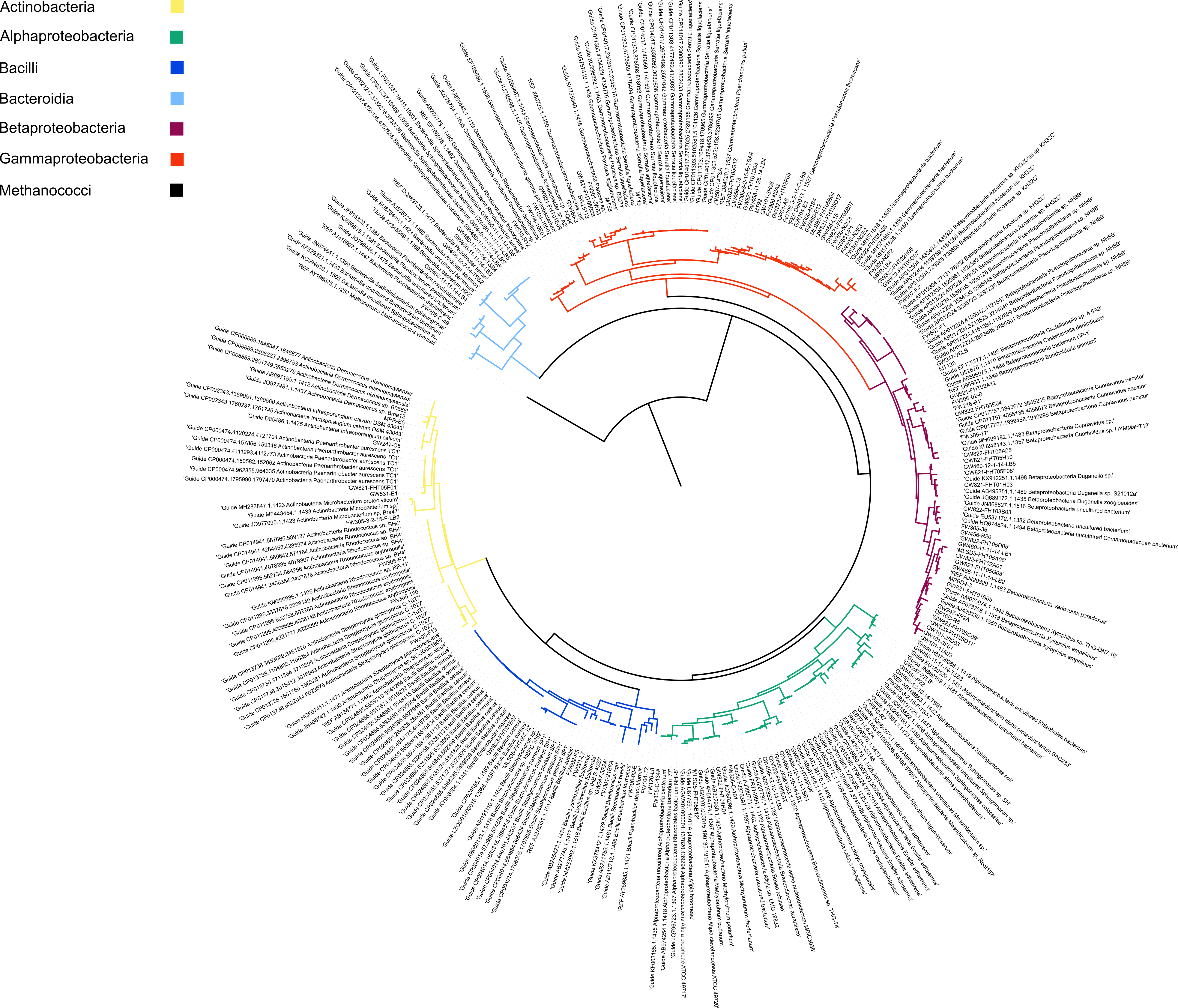

Supplement: Supplementary Figure 1 — Phylogenetic tree of isolate sequences, guide sequences and reference sequences. The phylogenetic tree forms the basis for the phylogenetic tree in Figure 1. Label colors indicate the phylogenetic origin of each sequence by class. REF and Guide indicates reference organism and were inserted for guidance and not used in this study. A complete list of the FASTA sequences can be found in Supplementary Information. [file Image_1.PNG]

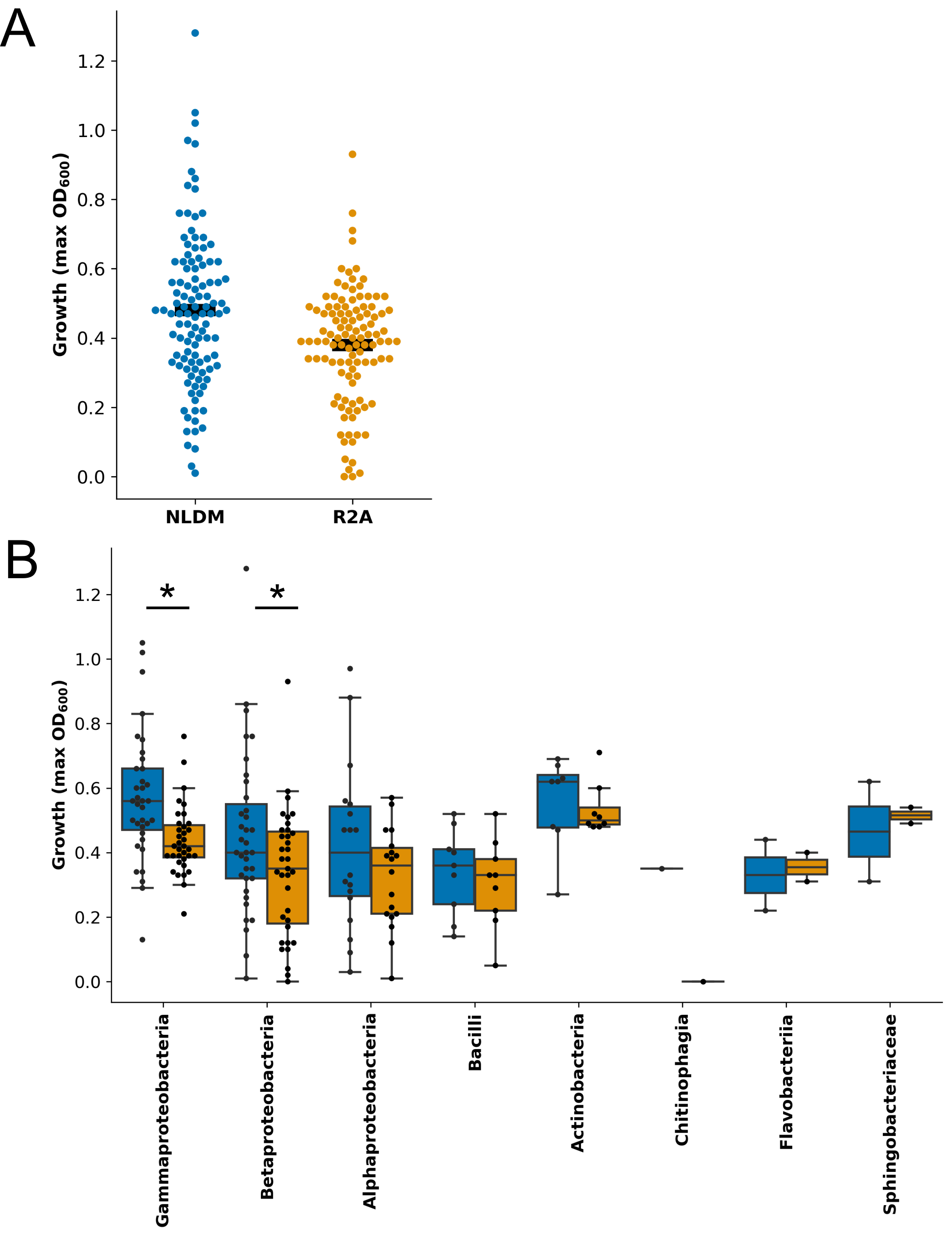

Supplement: Supplementary Figure 2 — Growth of isolates grown in R2A and NLDM. (A) Average (n = 2 or n = 3) growth (as measured as the highest observed OD600 minus starting OD600) of each isolate grown on NLDM (in blue) and R2A (yellow). Black bars represent the average for each medium type. (B) Average (n = 2 or n = 3) growth (as measured as the highest observed OD600 minus starting OD600) of each isolate grown on NLDM (in blue) and R2A (yellow) per phylogenetic class. *Indicate that growth was significantly (P < 0.05) higher in NLDM compared to R2A (pairwise t-test). [file Image_2.PNG]

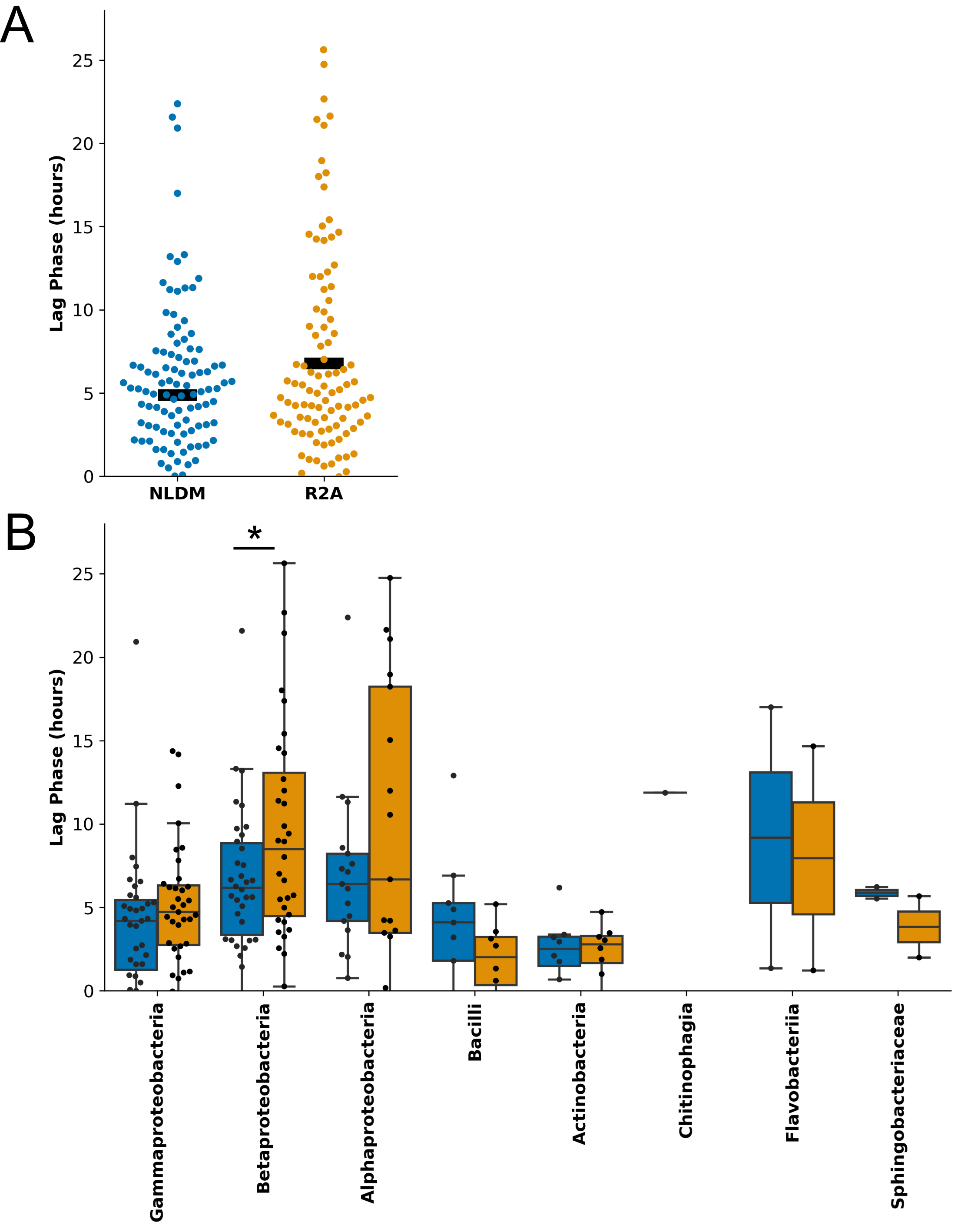

Supplement: Supplementary Figure 3 — Lag phase of isolates grown in R2A and NLDM. (A) Average (n = 2 or n = 3) lag phase of each isolate grown on NLDM (in blue) and R2A (yellow). Black bars represent the average for each medium type. (B) Average (n = 2 or n = 3) lag phase of each isolate grown on NLDM (in blue) and R2A (yellow) per phylogenetic class. *Indicate that the lag phase was significantly (P < 0.05) shorter in NLDM compared to R2A (pairwise t-test). [file Image_3.PNG]

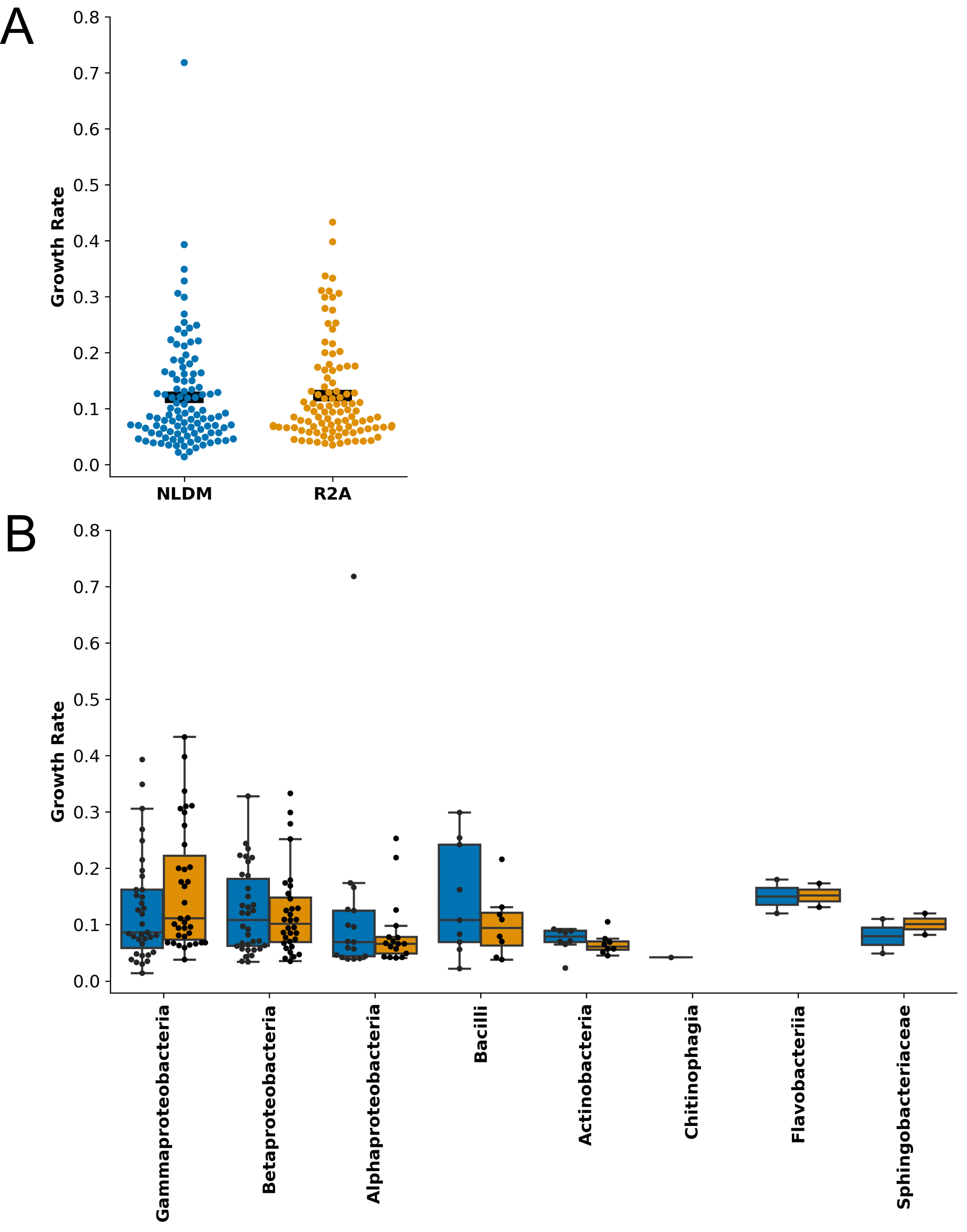

Supplement: Supplementary Figure 4 — Growth rates of isolates grown in R2A and NLDM. (A) Average (n = 2 or n = 3) growth rate of each isolate grown on NLDM (in blue) and R2A (yellow). Black bars represent the average for each medium type. (B) Average (n = 2 or n = 3) growth rates of each isolate grown on NLDM (in blue) and R2A (yellow) per phylogenetic class. [file Image_4.PNG]

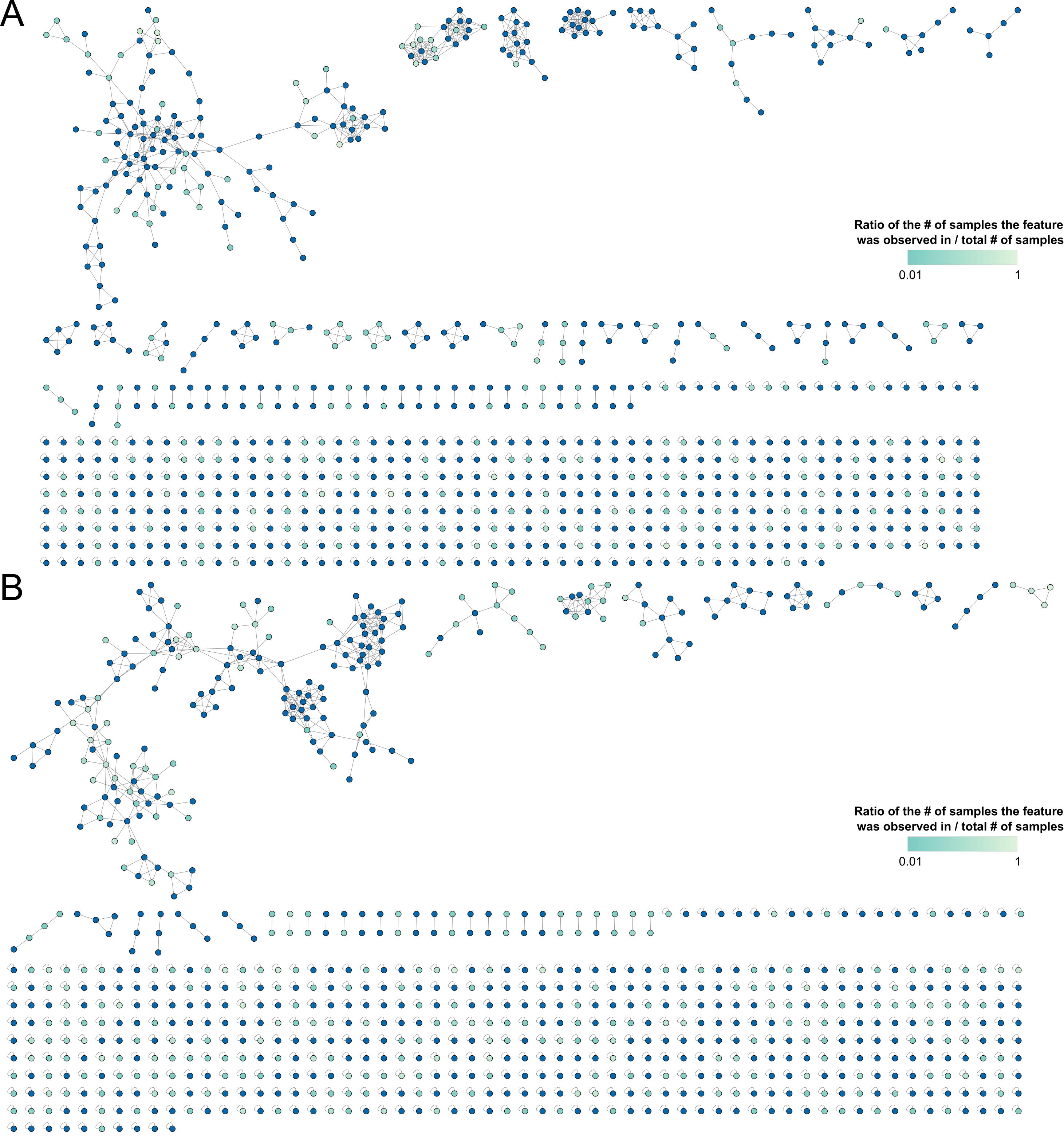

Supplement: Supplementary Figure 5 — Feature-based molecular networking. Identification of “produced” metabolites by comparing features observed in uninoculated medium vs. in isolate spent medium. Feature-based molecular networks with features detected in positive (A) and negative (B) polarity were colored to illustrate signals coming from metabolites produced by the isolates in this study. Features observed in less than 16% of NLDM uninoculated controls were colored from dark to light based on the ratio (0.01–1) of the number of samples of isolate spent medium the feature was observed in divided by the total number of isolate spent medium samples, with a ratio of 1 indicating that the feature was observed in all isolate spent medium samples. [file Image_5.PNG]
